# GhMPK9‐GhRAF39_1‐GhWRKY40a Regulates the GhERF1b‐ and GhABF2‐Mediated Pathways to Increase Cotton Disease Resistance

**DOI:** 10.1002/advs.202404400

**Published:** 2024-06-06

**Authors:** Xinyue Mi, Weixi Li, Chuan Chen, Huijuan Xu, Guilin Wang, Xuanxiang Jin, Dayong Zhang, Wangzhen Guo

**Affiliations:** ^1^ State Key Laboratory of Crop Genetics & Germplasm Enhancement and Utilization Engineering Research Center of Ministry of Education for Cotton Germplasm Enhancement and Application Nanjing Agricultural University Nanjing 210095 China

**Keywords:** Gossypium hirsutum, phosphorylation, Raf‐like kinase, transcription factor, Verticillium wilt

## Abstract

Mitogen‐activated protein kinase (MAPK) cascade is the center of plant signal transduction system that amplify immune signals into cellular responses by phosphorylating diverse substrates. The MAPK cascade consisting of MAPK kinase kinases (MAPKKKs), MAPK kinases (MAPKKs), and MAPKs is well characterized in plants, in which Raf‐like kinases are generally regarded as MAPKKKs. However, it is rarely reported that Raf‐like MAPKKKs function as middle regulators to link MAPK and its downstream transcription factors in plant immunity. Verticillium wilt, caused by the soil‐borne vascular fungus *Verticillium dahliae*, is a serious disease in many plants, including cotton. The previous studies showed that GhMPK9 (a MAPK) is involved in the response to Verticillium wilt. Here, the Raf‐like kinase GhRAF39_1 is reported as helper regulates the phosphorylation of WRKY transcription factor GhWRKY40a by GhMPK9. The phosphorylated GhWRKY40a can further activate the transcription of *GhERF1b* to up‐regulate defense‐related genes while inhibit the transcription of *GhABF2* to regulate the stomatal opening, thus improving the resistance to Verticillium wilt in cotton. This study reveals a new signaling module of GhMPK9‐GhRAF39_1‐GhWRKY40a to regulate *GhERF1b*‐ and *GhABF2*‐mediated defense responses, which triggers plant defense against Verticillium wilt.

## Graphical abstract

When *Verticillium dahliae* attacks cotton plants, GhMPK9‐GhRAF39_1‐GhWRKY40a in sequential phosphorylation activates GhWRKY40a. The phosphorylated GhWRKY40a has the dual functions, which enhances the transcriptional activity of *GhERF1b* to promote the expression levels of PR genes, and suppresses the transcriptional activity of *GhABF2* to regulate the leaf stomata opening causing the loss of leaf moisture, thereby increasing cotton resistance to *V. dahliae*.

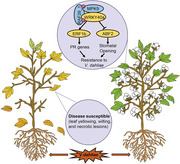

## 1 Introduction

The process of which pathogen infestation triggers a defense response in plants involves a complex and ingenious signaling system, and the mitogen‐activated protein kinase (MAPK) cascade pathway is located at the center of this system.^[^ ^1^ ^]^ A typical MAPK cascade consists of a three‐tiered system, one MAPK kinase kinase (MAPKKK), one MAPK kinase (MAPKK), and one MAPK to transduce signals through sequential phosphorylation. The activated MAPKs further phosphorylate the specific downstream substrates, leading to the priming of cellular responses.^[^ ^2^ ^]^ There are two types MAPKKKs, MEKK‐like kinases and Raf‐like kinases. MEKK‐like kinases have been well characterized, which positively regulate the downstream MAPKK‐MAPK module as typical MAPKKKs. In *Arabidopsis thaliana*, the pattern recognition receptor flg22 activates two cascade signaling pathways, MEKK1‐MKK4/5‐MPK3/6 and MEKK1‐MKK1/2‐MPK4, to induce the expression of relevant defense genes or camalexin biosynthesis in response to the fungus, with the former playing a positive regulatory role and the latter playing a negative regulatory role in the defense response.^[^ ^3^ ^]^ However, the function of Raf‐like kinases remains to be understood. Several studies show that Raf‐like kinases negatively regulate the MAPK cascade. CTR1, an original member of the plant Raf‐like kinase family, negatively regulates MKK9‐MPK3/6 cascade and directly phosphorylates EIN2 in ethylene‐signal conduction.^[^ ^4^ ^]^ EDR1 negatively regulates MKK4/MKK5‐MPK3/MPK6 to fine‐tune plant innate immunity.^[^ ^5^ ^]^ In rice, OsMPK6 has been shown to phosphorylate a Raf‐like kinase ENHANCED DISEASE RESISTANCE 1 (OsEDR1), leading to increased OsMKK10‐2 activity and resistance to the bacterial pathogen *Xanthomonas oryzae* pv. *oryzicola* (*Xoc*).^[^ ^6^ ^]^ A recent study suggests that AtMPK3 phosphorylates AtMAPKKK5 to enhance MAPK cascade activation and disease resistance in *edr1* mutants.^[^ ^7^ ^]^ Despite these reports, the function of Raf‐like kinases in MAPK cascade remains to be determined.

The identification of MAPK substrates is another key point to understand the functions and underlying mechanisms of MAPK signaling. Supporting evidence shows many phosphorylated substrates downstream of *Arabidopsis thaliana* MPK3 and MPK6 play important roles in plant immunity, response to abiotic stresses, and plant growth and development.^[^ ^8^ ^]^ 1‐Aminocyclopropane‐1‐carboxylic acid synthase (ACS), as the first plant MAPK substrate, is phosphorylated by MPK3/MPK6 to stabilize ACS6 protein in vivo, leading to increased ACS6 activity and elevated ethylene production.^[^ ^9^ ^]^ WRKY33 is required for MPK3/MPK6‐induced camalexin biosynthesis.^[^ ^10^ ^]^ It was also found that MPK3/MPK6 can phosphorylate WRKY33 to regulate the expression of ACS2 and ACS6 in response to pathogen infection by directly binding to the ACS2/ACS6 promoter in vivo.^[^ ^11^ ^]^ In addition to regulating hormone biosynthesis, MPK3/MPK6 can also activate defense gene expression by directly phosphorylating downstream transcription factors to control immune signal transduction. In *Arabidopsis thaliana*, the phosphorylation of ethylene response factors (such as ERF6 and ERF104) by MPK3/MPK6 promotes their protein stabilization, which further activates the expression of several defense‐related genes such as PDF1.1, PDF1.2a, PDF1.2b, ChiB, and HEL.^[^ ^12^ ^]^ VIP1 (bZIP transcription factor) is phosphorylated by MPK3, resulting in VIP1 relocation from the cytoplasm to the nucleus and activation of pathogenesis‐related (PR) genes.^[^ ^13^ ^]^ In rice, OsMKK10‐2 has been reported to activate OsMPK3 and OsMPK6 for enhanced resistance to the bacterial streak pathogen *Xoc* and tolerance to drought stress.^[^ ^14^ ^]^ OsMKK10‐2 physically interacts with OsWRKY31 that is phosphorylated by OsMPK3, OsMPK4, and OsMPK6. Phosphorylated OsWRKY31 has elevated DNA‐binding activity and confers enhanced resistance to the rice blast pathogen *Magnaporthe oryzae*.^[^ ^15^ ^]^ In a word, the identification of novel substrates of MAPK will help us understand how a limited number of MAPKs can play important roles in a variety of biological processes in plants.

Pathogenic fungi can induce disease symptoms in many plant species and cause severe agricultural and economic losses.^[^ ^16^ ^]^ Verticillium wilt, caused by the soil‐borne vascular fungus *Verticillium dahliae*, is a serious disease in many plants. The *V. dahliae* enters the plant through the roots and multiplies massively in the vascular system, eventually exhibiting the symptoms of chlorosis and wilting.^[^ ^17^ ^]^ Cotton (*Gossypium* spp.) is one of the most important economic crops worldwide, and Verticillium wilt is the major limitation on cotton yield and quality globally.^[^ ^18^ ^]^ The immune signaling pathway mediated by MAPK cascade plays a key role in regulating resistance to pathogens in cotton. Overexpressing cotton *GhMPK2* in tobacco enhanced the resistance to tobacco mosaic virus and Fusarium wilt,^[^ ^19^ ^]^ and overexpression of *GhMPK16* in *Arabidopsis thaliana* enhanced its resistance to *Pseudomonas aeruginosa*.^[^ ^20^ ^]^ The MKK members of the MAPK cascade pathway play a dual role in fine‐tuning cotton resistance to Verticillium wilt. *GhMKK4*, *GhMKK6*, and *GhMKK9* positively regulate the Verticillium wilt resistance, while *GhMKK10* negatively regulates the resistance.^[^ ^21^ ^]^ Silencing *GhMKK2* impaired significantly Verticillium wilt resistance in cotton,^[^ ^22^ ^]^ and overexpression of *GhMKK5* in tobacco improved the tolerance to pathogenic infestation.^[^ ^23^ ^]^ The *GhMKK4*‐*GhMPK20*‐*GhWRKY40* cascade negatively regulates cotton resistance to Fusarium wilt.^[^ ^24^ ^]^ Overexpression of the scaffolding protein *GhMORG1* of the *GhMKK6*‐*GhMPK4* cascade enhances resistance to Fusarium wilt in cotton.^[^ ^19^ ^]^ The *GhMKK2*‐*GhNTF6*‐*GhMYC2* cascade increases resistance to Fusarium wilt by up‐regulating the expression of flavonoid biosynthesis‐related genes.^[^ ^25^ ^]^ These studies show that the MAPK cascade plays an important role in plant resistance to pathogens in cotton. However, most of them merely concentrate on the MAPKKK‐MAPKK‐MAPK cascade or the MAPK phosphorylate downstream transcription factors. Few studies show that Raf‐like kinase can be used as a middle regulator between MAPK and its downstream transcription factors in plant immunity.

In our previous studies of cotton responding to Verticillium wilt, a total of 28 candidate MAPK family members were genome‐wide identified and *GhMPK9* was regarded as a key disease‐resistant gene to Verticillium wilt.^[^ ^26^ ^]^ Here, we revealed a new regulatory network downstream of *GhMPK9* to regulate cotton disease resistance. *GhMPK9* is an essential gene in cotton. Overexpression of *GhMPK9* activates defense response to enhance the resistance to Verticillium wilt in cotton, while suppressing *GhMPK9* causes male and female sterility in cotton and fails to produce progeny. GhRAF39_1 and GhWRKY40a are identified as potential substrates downstream of GhMPK9 to form protein complexes, however, GhMPK9 could not phosphorylate directly GhWRKY40a. GhRAF39_1 as helper functions in phosphorylation of GhWRKY40a by GhMPK9. The phosphorylated GhWRKY40a has the dual functions, which activates the transcription of *GhERF1b* to initiate the downstream PR genes and inhibits the transcription of *GhABF2* to keep the stomatal opening, thereby improving the resistance of cotton to Verticillium wilt. Taken together, we reveal that a novel GhMPK9‐GhRAF39_1‐GhWRKY40a module functions as a positive regulator in the resistance of cotton to Verticillium wilt by modulating *GhERF1b*‐mediated upregulation of PR genes and *GhABF2*‐mediated stomatal opening.

## 2 Results

### 2.1 *GhMPK9* is a Critical Gene in Plant Immunity and Reproductive Development in Cotton

**Figure 1:**
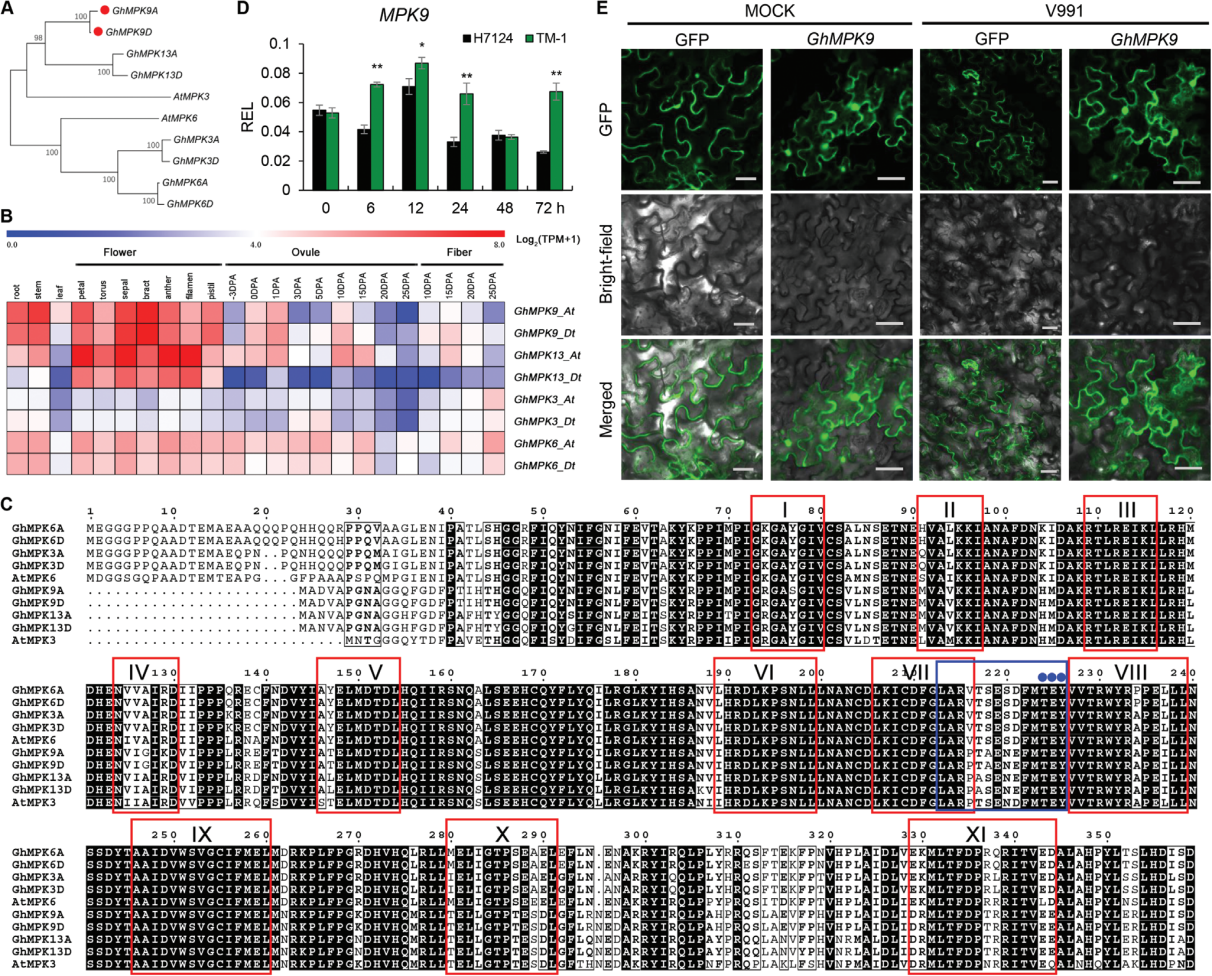
Structure, expression, phylogenetic relationship, and subcellular localization of GhMPK9. A) The phylogenetic relationship of *GhMPK9* and its homologous genes in *Gossypium hirsutum* (Gh) and *Arabidopsis thaliana* (At). B) The expression patterns in different tissues and organs for *GhMPK9* homologous genes. Colored squares indicate expression values of the selected genes from 0 (blue) to 8 (red) normalized by Log_2_(TPM+1). DPA: day post anthesis. C) Protein sequence alignments of GhMPK3A/D, GhMPK6A/D, GhMPK9A/D, GhMPK13A/D, and AtMPK3/6. Identical amino acids are shaded in black. The conserved subdomains are shown with red boxes (I‐XI). The TEY motif is marked by blue dots at the top of the sequences. D) Expression levels of *MPK9* in V991‐treated roots of cotton seedlings at different time points in *G. hirsutum* acc. TM‐1 and *G. barbadense* cv. H7124. The expression levels were validated by RT‐qPCR. The relative expression levels (REL) are normalized to those of *Histone3*. E) Subcellular localization of GhMPK9‐GFP protein under normal and V991 conditions. GFP fluorescence was observed after transiently expressing GhMPK9‐GFP protein in tobacco epidermal cells. Bars = 50 µm. Data are presented as means ± SD in (D), *n* = 9, statistical analyses are performed using Student's *t* test: **, *P* < 0.01, *, *P* < 0.05.

In previous studies, we identified genome‐wide MAPK family members in cotton and found a gene named *GhMPK9* was involved in the response of cotton to Verticillium wilt.^[^ ^26^ ^]^ To investigate how *GhMPK9* regulates the resistance, we cloned the full‐length cDNA of *GhMPK9_A/D* from *Gossypium hirsutum* L. acc TM‐1, which was homologous with the sequence of *GhMPK3_A/D*, *GhMPK6_A/D*, and *GhMPK13_A/D* in the cotton genome. Phylogenetic analysis showed that GhMPK9 and GhMPK13 were homologous to AtMPK3, while GhMPK3 and GhMPK6 were homologous to AtMPK6 (**Figure** **1**A). Expression analysis confirmed that both *GhMPK9* and *GhMPK13* were highly expressed in floral organs, but *GhMPK9* expression was higher than that of *GhMPK13* in roots and stems (Figure 1B). Multiple sequence alignments indicated that there were amino acid differences among them in 11 conserved subdomains and an activation loop with TEY motif (Figure 1C). The reverse transcription quantitative polymerase chain reaction (RT‐qPCR) analysis showed there were no obvious differences in expression levels of *MPK9* homologs between resistant cultivar (*G. barbadense* L. cv. H7124) and susceptible accession (*G. hirsutum* L. acc TM‐1) under normal condition, but the expression of *MPK9* was significantly higher in susceptible accession TM‐1 than resistant cultivar H7124 at 6, 12, 24, and 72 h post inoculation (hpi) by *V. dahliae*, suggesting *MPK9* was basal and vital in plant immunity of different cotton species with different immune responses (Figure 1D). Subcellular localization in *N. benthamiana* leaves epidermis suggested that GhMPK9 was a nucleus and plasma membrane localized protein under normal and inoculated V991 conditions (Figure 1E). Virus induced gene silencing (VIGS) analysis indicated that silencing *GhMPK9* caused the cotton susceptibility to *V. dahliae*, which were consistent with previous study that *GhMPK9* may participate in the cotton defense response to *V. dahliae* infecting in roots (Figure S1, Supporting Information).^[^ ^26^ ^]^

To investigate the biological function of *GhMPK9*, we produced RNA interfering (RNAi) lines by Agrobacterium‐mediated genetic transformation system in cotton. The DNA and RNA level detection showed *GhMPK9* was significantly suppressed in five RNAi lines (Figure S2A,B, Supporting Information). Unfortunately, all *GhMPK9*‐RNAi lines failed to produce bolls and seeds for reproduction. We found the stamens of the *GhMPK9*‐RNAi lines developed abnormally and could not produce fertile pollen (Figure S2C, Supporting Information). Thus, we pollinated the fertile pollen from wild‐type (WT) to the *GhMPK9*‐RNAi lines, while it was still unsuccessful, indicating that the pistils were also abnormally developed (Figure S2D, Supporting Information). These results indicate that *GhMPK9* plays an important role in the development of reproductive organs in cotton.

### 2.2 Overexpression of *GhMPK9* Activates Plant Immune Responses to Enhance Resistance to Verticillium wilt

**Figure 2:**
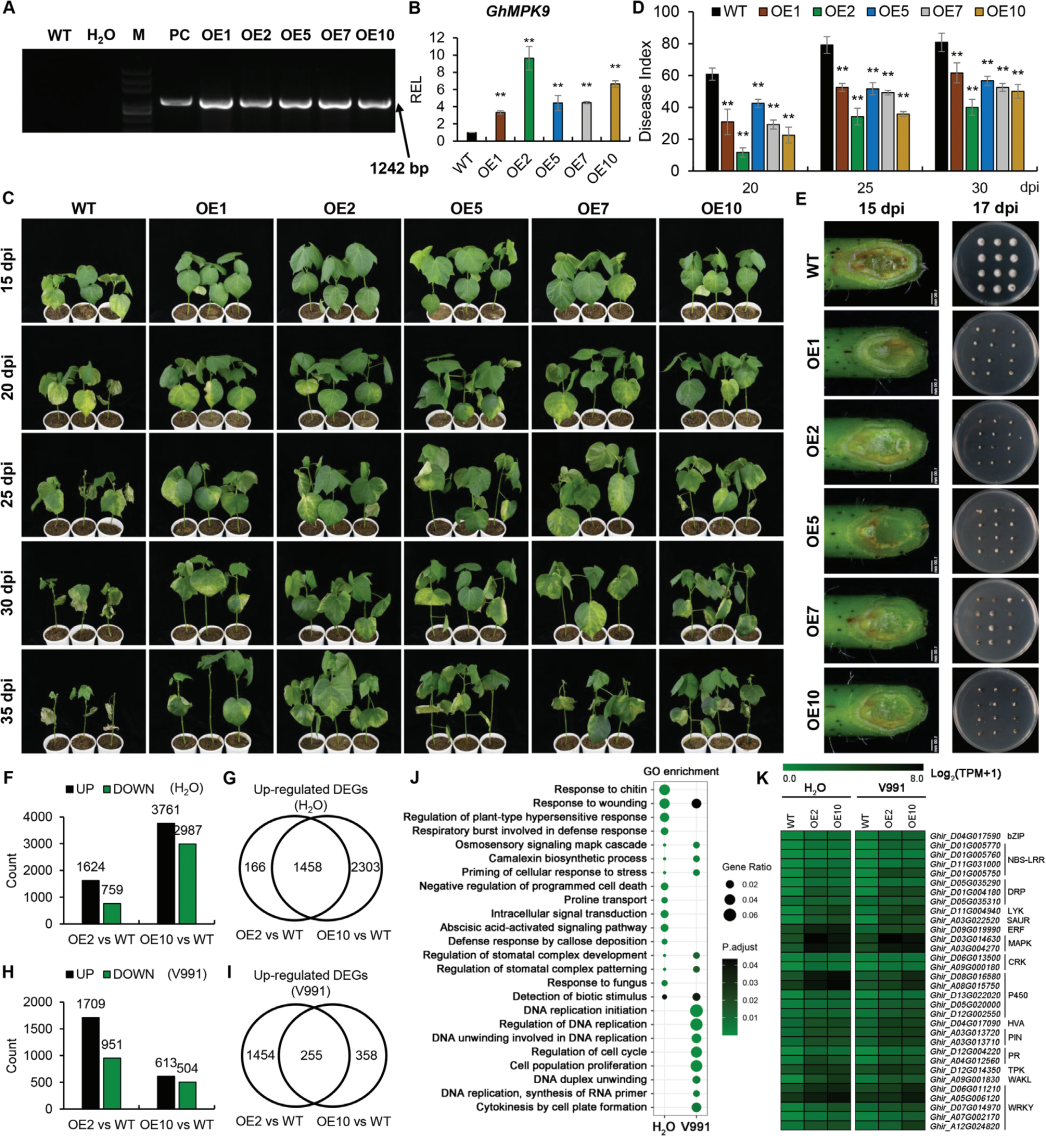
Overexpression of *GhMPK9* activates cotton immune responses and enhances resistance to Verticillium wilt. A) DNA detection of *GhMPK9* transgenic lines by PCR analysis. PC: positive control; WT: wild‐type. OE1, OE2, OE5, OE7, and OE10: five *GhMPK9* transgenic overexpression lines. B) The relative expression level of *GhMPK9* in wild‐type and transgenic lines. Expression levels were normalized to those of *Histone3*. C) Plant phenotypes of OE lines and WT after inoculating with *V. dahliae* at different time points. Three‐week‐old seedlings of WT and OE lines were inoculated with *V. dahliae* and replanted in soil, with at least 30 plants for each line, and photographed from 15 to 35 dpi. D) The disease index of WT and OE plants at 25, 30, and 35 dpi. E) V991 hyphal growth observation and fungal recovery experiments for WT and OE plants at 15 dpi. Stem sections were cut and incubated on potato dextrose agar plates at 25 °C. Photographs were taken after two days. F,H) Number of up‐ and down‐regulated DEGs between WT and OE2/OE10 under normal (F) and V991(H) conditions. The green column represents the downregulated genes and the dark column represents the upregulated genes. G,I) Venn diagram showing overlap among up‐regulated DEGs between the WT and OE2/OE10 under normal (G) and V991(I) conditions. J) Gene Ontology (GO) analysis of the overlapped DEGs between WT and OE2/OE10. K) Heatmap of the expression levels of disease‐resistant genes in WT, OE2, and OE10 under normal and V991 conditions. Colored squares indicate expression values of the selected genes from 0 (green) to 8 (black) normalized by Log_2_(TPM+1). Data are presented as means ± SD in (B and D), *n* = 9 (B), *n* = 30 (D), statistical analyses are performed using Student's *t* test: **, *P* < 0.01.

We further generated *GhMPK9* overexpression lines (OE) driven by 35S promoter via Agrobacterium‐mediated genetic transformation system in cotton. In contrast to the *GhMPK9*‐RNAi lines, the fertility of *GhMPK9*‐OE lines was not affected, and the quality and yield of the mature fibers had no obvious difference compared to WT (Table S1, Supporting Information). Then, we used five stable overexpression transgenic lines, OE1, OE2, OE5, OE7, and OE10, in which the transcripts level of *GhMPK9* was significantly higher than in WT, to further elucidate the function of *GhMPK9* in response to Verticillium wilt (**Figure** **2**A,B). After growing to the two‐leaf stage, the overexpression and wild‐type seedlings were inoculated with V991. Subsequent observations found that WT showed obvious cotyledon wilt and partial yellowing of the true leaves from 15 day post inoculation (dpi), with more and more true leaves yellowing and falling off with time (Figure 2C; Figure S2E, Supporting Information). However, the *GhMPK9* overexpression transgenic lines exhibited lighter disease symptoms at the same time compared to WT (Figure 2C; Figure S2E, Supporting Information). The disease index of five OE lines were all significantly lower than that of wild‐type seedlings at 20, 25, 30 dpi (Figure 2D). In stems, the accumulation of V991 mycelium in WT was much more than OE lines at 15 dpi (Figure 2E). Meanwhile, the stems of wild‐type and overexpression plants were intercepted into stem sections of ≈5 mm to culture on PDA solid medium for *V. dahliae* recovery experiments. After two days, the fungal recovery was obviously higher in WT than in all OE lines (Figure 2E). Besides, we observed that in different OE lines, the higher expression of GhMPK9 is, the stronger disease resistance is, indicating a dose‐dependent association between the expression of *GhMPK9* and the resistance to *V. dahliae*. Taken together, the overexpression of *GhMPK9* improves the resistance of cotton to Verticillium wilt.

Next, we selected two highly overexpressed lines, OE2 and OE10, to compare their transcriptome differences under normal and V991 conditions. Compared to WT, there were 1624 up‐regulated differentially expressed genes (DEGs) in OE2 and 3761 up‐regulated DEGs in OE10 with 1458 overlapped DEGs under normal condition (Figure 2F,G; and Data S1, Supporting Information). Under V991 condition, we identified 1709 up‐regulated DEGs in OE2 and 613 up‐regulated DEGs in OE10 with 255 overlapped DEGs (Figure 2H,I; and Data S1, Supporting Information). Gene Ontology (GO) enrichment analysis showed that these overlapped genes under both conditions were enriched in response to wounding, osmosensory signaling MAPK cascade, camalexin biosynthetic process, priming of cellular response to stress, detection of biotic stimulus, regulation of stomatal complex development and patterning (Figure 2J; and Data S2, Supporting Information). Many common disease‐resistant genes were significantly up‐regulated in OE lines under both normal and V991 conditions, such as WRKYs, ERFs, NBS‐LRRs, PRs, CRKs, P450s, and so on (Figure 2K). Collectively, overexpression of *GhMPK9* activates plant immune responses and regulates stomatal movement, which enhance resistance when *V. dahliae* attacks.

### 2.3 *GhMPK9* and *GhWRKY40a* Show the Co‐Expression Profiles and their Encoding Proteins Interacts Physically

**Figure 3:**
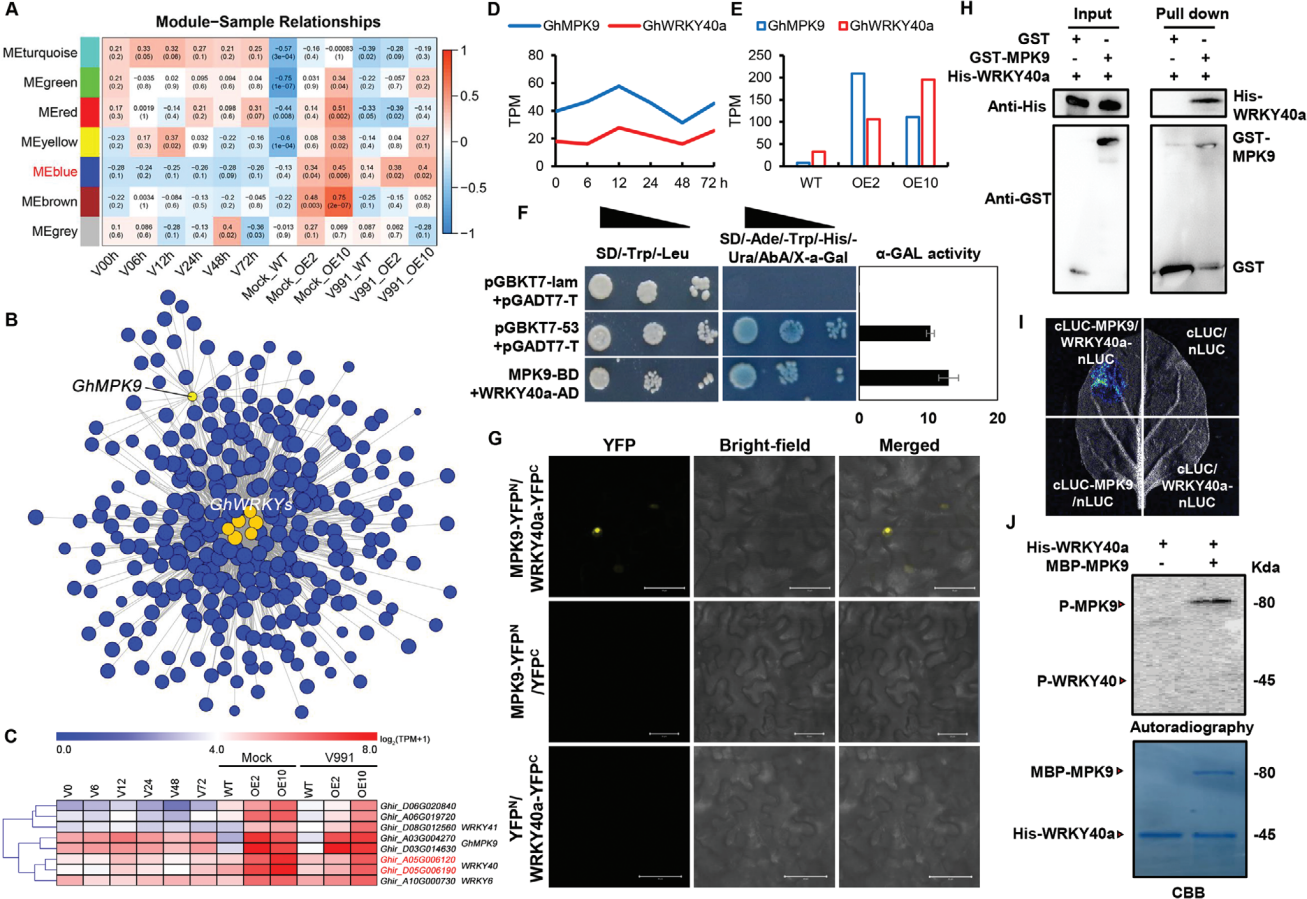
*GhMPK9* and *GhWRKY40a* co‐expresses and their encoding protein interacts physically. A) Co‐expression modules information. Each row in the table corresponds to a consensus module, and each column to sample with different time point of hour post inoculation. Numbers in the table indicate the correlations of the corresponding module and sample, with the *p*‐values printed below the correlations in parentheses. The table is color coded by correlation according to the color legend. The MEblue represents genes co‐expressed with *GhMPK9*. B) Network analysis of *GhMPK9* based on weighted correlation network analysis. The yellow circle represents *GhMPK9*; the orange circle represents WRKYs co‐expressed with *GhMPK9*. C) Heatmap of the expression patterns for co‐expressed WRKYs induced by *V. dahliae*. Colored squares indicate expression levels of the selected genes from 0 (blue) to 6 (red) normalized by Log_2_(TPM+1). D,E) Expression patterns of *GhMPK9* and *GhWRKY40a* in V991‐treated at different time points (D) and transgenic lines (E). The expression levels were normalized by TPM using RNA‐seq data. F) Y2H assays and α‐galactosidase activity for GhMPK9‐GhWRKY40a interaction. Activity was determined by incubating the yeast to a concentration OD_600_ of ≈0.5. Data are means (±SD) of three biological replicates. G) BiFC assays for GhMPK9‐nYFP and GhWRKY40a‐cYFP in tobacco epidermal cells. Bars = 50 µm. H) GST‐GhMPK9 protein pulls down His‐GhWRKY40a protein in vitro. The anti‐His antibody is used to detect the output protein. I) LCI experiments of cLUC‐GhMPK9 and GhWRKY40a‐nLUC in tobacco leaves. J) In vitro phosphorylation experiments of GhMPK9 and GhWRKY40a. The phosphorylation reactions using MBP‐GhMPK9 as the kinase and His‐GhWRKY40a as the substrate were reacted with 10 µCi [γ−^32^P]‐ATP at 30 °C for 30 min. The phosphorylated proteins were detected by autoradiography. Protein inputs were detected by Coomassie brilliant blue (CBB) staining. Data are presented as means ± SD in (F), *n* = 3.

Given that genes with similar expression patterns might be relevant in function or co‐regulated in pathway, we performed the weighted gene co‐expression network analysis (WGCNA) analysis in 1458 up‐regulated DEGs in OE lines to identify the key regulatory genes downstream of GhMPK9. Due to the insufficient expression diversity of these 1458 genes in OE and WT samples, we added transcriptome data for different time points of V991 infection in upland cotton from the previous study.^[^ ^27^ ^]^ To mine key candidate DEGs which were not only up‐regulated in OE lines but also co‐expressed with *GhMPK9* in response to *V. dahliae*, we identified six modules of co‐expressed genes by (WGCNA), for which genes in color‐coded modules ranged in size from 42 to 562 (**Figure** **3**A; Figure S3A–C and Data S3, Supporting Information). Of them, *GhMPK9* and other 468 genes in MEblue showed peak expression at 12 h post inoculation (Figure S3D, Supporting Information), and they were significantly enriched in activation of MAPK activity involved in osmosensory signaling pathway, priming of cellular response to stress, and camalexin biosynthetic process (Data S3, Supporting Information). According to the correlation between genes in MEblue, we constructed a co‐expression network of *GhMPK9* and showed several WRKY transcription factors within it (Figure 3B), which were widely considered as the downstream substrates of MAPK.^[^ ^28^ ^]^ By comparing and clustering the expression profiles of these WRKYs in different RNA‐seq samples, we found *GhWRKY40a* was most closely clustered with *GhMPK9* in expression (Figure 3C–E). Finally, we selected *GhWRKY40a* as a key candidate gene for subsequent analysis. Previous studies demonstrated that WRKY40, as a pathogen‐induced transcription factor, formed protein complexes with its homologous genes WRKY18 and WRKY60 in *Arabidopsis thaliana*.^[^ ^29^ ^]^ In cotton, we found five pairs of WRKY40 homologous genes and *GhWRKY40a* had similar tissue expression patterns as *GhMPK9* (Figure 1C; Figure S4, Supporting Information). The yeast two hybrid (Y2H) assays as well as α‐galactosidase activity assay showed that GhMPK9 interacted with GhWRKY40a (Figure 3F). Bimolecular fluorescence complementation (BiFC) by transiently co‐expressing both proteins in tobacco leaf epidermal cells showed that strong YFP fluorescence signals were observed in the nucleus (Figure 3G). GST pull‐down assays further confirmed that His‐GhWRKY40a proteins were pulled down by GST‐GhMPK9, but not by GST (Figure 3H). The interaction signals between GhMPK9 and GhWRKY40a proteins were also detected in luciferase complementation imaging (LCI) experiments (Figure 3I). These results confirmed that GhMPK9 interacted physically with GhWRKY40a. However, to our surprise, GhMPK9 could not phosphorylate directly GhWRKY40a. There was no phosphorylation band visualized by autoradiography in His‐GhWRKY40a after incubating with MBP‐GhMPK9 (Figure 3J).

### 2.4 Phospho‐Proteome Analysis Reveals GhRAF39_1 as a Phosphorylated Substrate of GhMPK9

**Figure 4:**
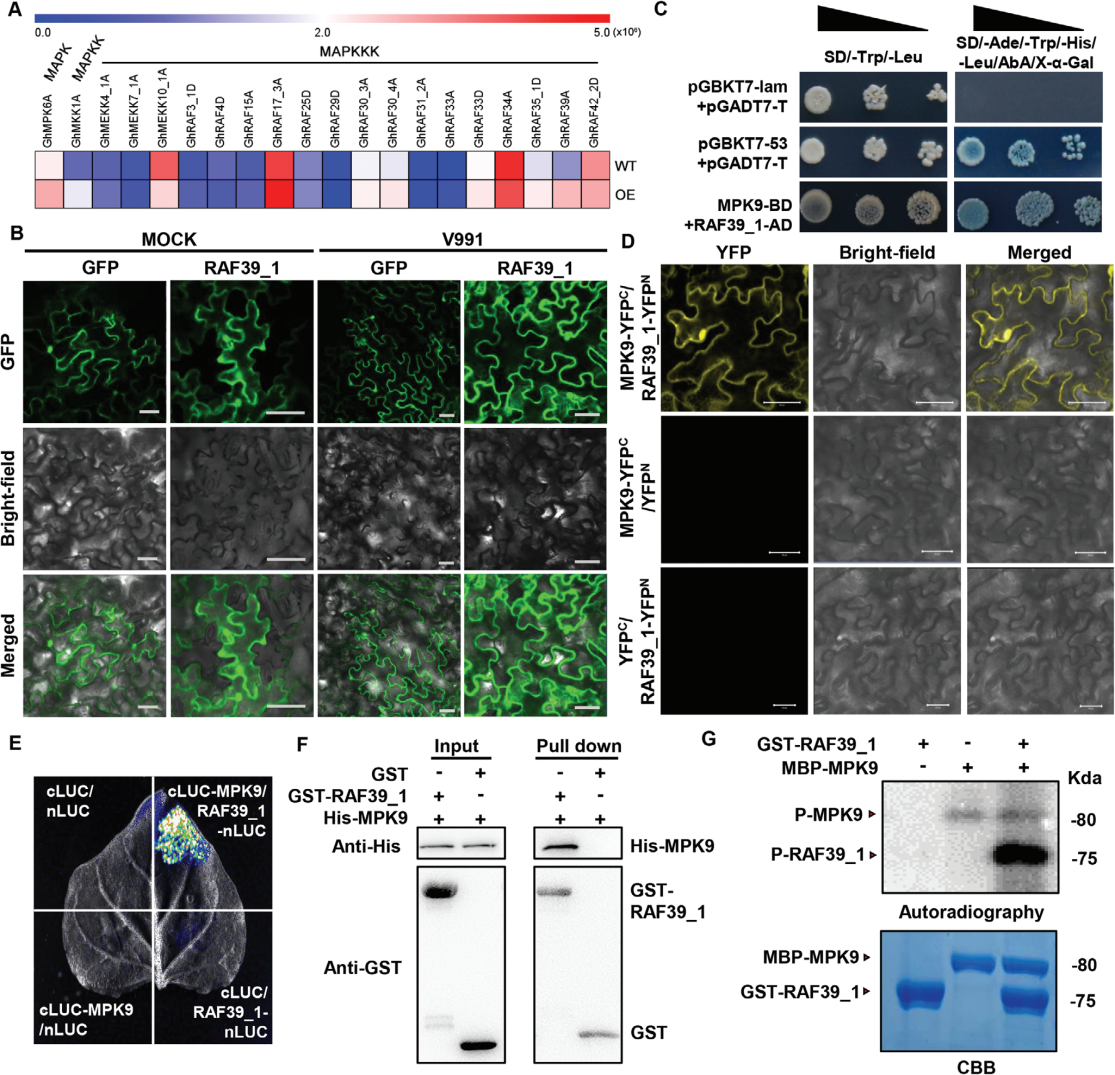
GhMPK9 physically interacts with GhRAF39_1 and phosphorylates GhRAF39_1. A) Heatmap of the quantification of differentially phosphorylated MAPKs, MAPKKs, and MAPKKKs. Colored squares indicate protein abundances of the selected genes from 0 (blue) to 5 x 106 (red) by label‐free quantification. B) Subcellular localization of GhRAF39_1 under normal and V991 conditions. C) The interaction of GhMPK9 with GhRAF39_1 validated by Y2H assays. The BD‐53/AD‐T was used as the positive control, and the BD‐Lam/AD‐T was used as the negative control. D) BiFC assays between GhMPK9‐cYFP and GhRAF39_1‐nYFP in tobacco epidermal cells. Bars = 50 µm. E) Luciferase complementation imaging assays detecting the interaction of GhMPK9 with GhRAF39_1. The cLUC‐GhMPK9 and GhRAF39_1‐nLUC vectors were co‐expressed in tobacco leaves and the LUC signal was imaged at 72 h post‐transfection. F) Glutathione S‐transferase (GST) pull‐down assays confirm the interaction of GhMPK9 with GhRAF39_1 in vitro. GST protein was used as the control. G) In vitro autophosphorylation assays to determine the phosphorylation of GST‐GhRAF39_1 by MBP‐GhMPK9. P‐GhRAF39_1: Phosphorylated protein of GhRAF39_1; P‐GhMPK9: Phosphorylated protein of GhMPK9. The phosphorylated proteins were detected by autoradiography. Protein inputs were detected by CBB staining.

Now that GhWRKY40a was not direct phosphorylation substrate of GhMPK9, we performed phospho‐proteome analysis using OE10 and WT to excavate downstream substrates of GhMPK9. Through liquid chromatography‐tandem mass spectrometry (LC‐MS/MS) analysis, we identified 4955 phosphorylation sites including 3852 serine sites, 989 threonine sites, and 114 tyrosine sites, and obtained a filtered data set of 4092 phospho‐peptides on 2312 phospho‐proteins (Data S4, Supporting Information). Of them, a total of 1923 stable phosphorylated proteins were identified in both OE10 and WT. Differential phosphorylation analyses discovered that numerous MAPK cascade genes had significant variances in phosphorylation levels, in which GhRAF39 were obviously activated (**Figure** **4**A; and Data S5, Supporting Information). Phylogenetic analysis showed there were six GhRAF39 homologs with highly conserved domains in cotton genome, and GhRAF39_1 showed the highest expression level in all tissues and organs (Figure S5A–C, Supporting Information). The motif prediction analysis suggested the potential phosphorylated site might be Ser27 in GhRAF39_1 (Figure S5D and Data S3, Supporting Information). Subcellular localization in *N. benthamiana* leaf epidermis showed that GhRAF39_1 localized in nucleus and plasma membrane under normal and inoculated V991 conditions (Figure 4B). Y2H assays demonstrated that GhMPK9 could interact with GhRAF39_1 (Figure 4C). Both BiFC and LCI experiments confirmed that GhRAF39_1 directly interacts with GhMPK9 in vivo (Figure 4D,E). To further clarify the kinase‐substrate relationships, we added the His and MBP tags to GhMPK9 as well as the GST tags to GhRAF39_1. In GST pull‐down assays, His‐GhMPK9 proteins were pulled down by GST‐GhRAF39_1 (Figure 4F). In phosphorylation experiments, there was a clear phosphorylation band visualized by autoradiography only in GST‐GhRAF39_1 incubating with MBP‐GhMPK9, indicating that GhMPK9 had kinase activity and activated GhRAF39_1 by phosphorylation in vitro (Figure 4G).

### 2.5 GhRAF39_1 as Helper Functions in Phosphorylation of GhWRKY40a by GhMPK9

**Figure 5:**
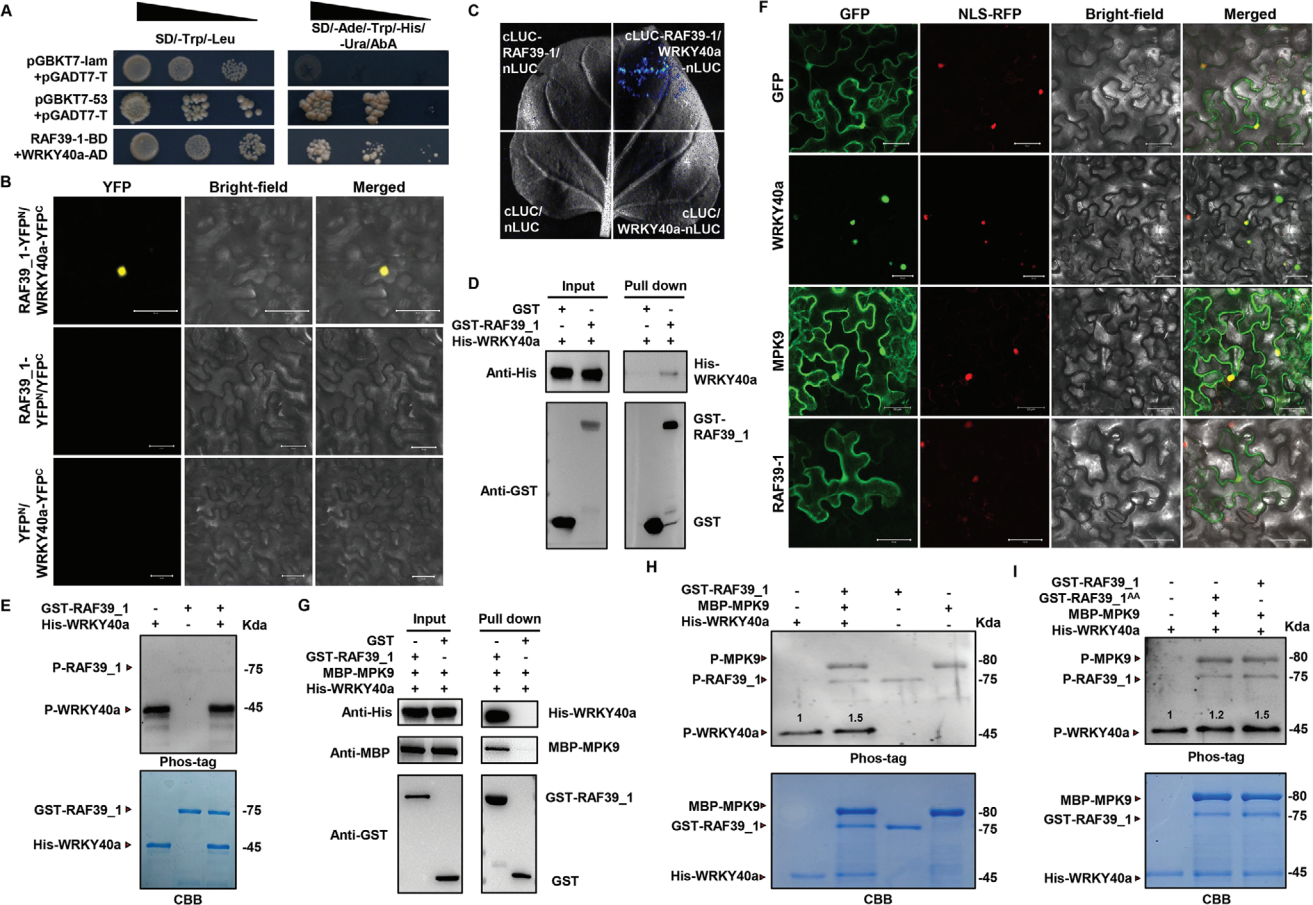
GhWRKY40a is phosphorylated by the GhMPK9‐GhRAF39_1 module. A) Y2H assays for GhRAF39_1‐GhWRKY40a interaction. B) BiFC assays for GhRAF39_1‐nYFP and GhWRKY40a‐cYFP in tobacco epidermal cells. Bars = 50 µm. C) LCI experiments of cLUC‐GhRAF39_1 and GhWRKY40a‐nLUC in tobacco leaves. D) GST‐GhRAF39_1 pulls down His‐GhWRKY40a in vitro. The anti‐His antibody is used to detect the output protein. E) Phosphorylation experiments of GhRAF39_1 and GhWRKY40a in vitro with biotin‐pendant Zn^2+^ phos‐tag (BTL‐111). CBB is used for proteins staining in phosphorylation assays. F) The subcellular localization of GhWRKY40a‐GFP, GhMPK9‐GFP, and GhRAF39_1‐GFP. Bars = 50 µm. G) Glutathione S‐transferase (GST) pull‐down assays confirm the interaction of GhMPK9 and GhWRKY40a with GhRAF39_1 in vitro. GST protein was used as the control. H) Phosphorylation experiments of GhMPK9, GhRAF39_1 and GhWRKY40a in vitro with biotin‐pendant Zn^2+^ phos‐tag (BTL‐111). CBB is used for proteins staining in phosphorylation assays. I) Phosphorylation experiments of GhRAF39_1 or GhRAF39_1^AA^, GhWRKY40a, and GhMPK9 in vitro with biotin‐pendant Zn^2+^ phos‐tag (BTL‐111). CBB is used for proteins staining in phosphorylation assays.

Considering there were interactions between GhMPK9 and GhRAF39_1 in a phosphorylated form (Figure 4), we further investigated the relationships between GhRAF39_1 and GhWRKY40a. The Y2H assays illustrated that GhRAF39_1 was able to interact with GhWRKY40a in yeast cells (**Figure** **5**A). The BiFC and LCI assays exhibited the interaction of GhRAF39_1 with GhWRKY40a in tobacco leaf epidermal cells (Figure 5B,C). The GST pull‐down experiment confirmed GST‐GhRAF39_1 pulled down a significant amount of His‐GhWRKY40a protein in vitro (Figure 5D). Through the Phos‐tag assay, we found that GhRAF39_1 could not phosphorylate GhWRKY40a individually, where the intensity of phosphorylated band in His‐GhWRKY40a incubated with GST‐GhRAF39_1 was same as the control band (Figure 5E). By coincidence, GhWRKY40a, GhMPK9 and GhRAF39_1 all localized in the nucleus according to the merged image of GhWRKY40a‐GFP, GhMPK9‐GFP, GhRAF39_1‐GFP and nuclear localization signal (NLS)‐mCherry signals (Figure 5F). This result implies that they may function as a whole. GST pull‐down assays further confirmed our hypothesis that His‐GhWRKY40a and MBP‐GhMPK9 proteins were pulled down by GST‐GhRAF39_1 (Figure 5G). These results demonstrated that GhWRKY40a could physically interact with GhMPK9 and its downstream GhRAF39_1 in the nuclear to form a ternary complex. Western blot analysis showed His‐GhWRKY40a was markedly phosphorylated when GST‐GhRAF39_1 was added with MBP‐GhMPK9, suggesting that GhMPK9‐GhRAF39_1 module activate the phosphorylation of GhWRKY40a (Figure 5H). Phosphorylation site analysis showed that GhRAF39_1 contained serine at positions 27, 34, 36, and 387 (Ser‐,^27^, Ser^34^, Ser^36^, and Ser^387^) and threonine at position 379, 381 and 386 (Thr^379^, Thr^381^, and Thr^386^) (Figure S5D, Supporting Information). When the Ser,^27^ Ser,^34^ Ser^36^, and Ser^387^ and Thr^379^, Thr^381^, and Thr^386^ of GhRAF39_1 was replaced by alanine (GhRAF39_1^AA^), the mutation diminished the phosphorylation of His‐GhWRKY40a incubated with GST‐GhRAF39_1^AA^ and MBP‐GhMPK9 (Figure 5I), indicating that the phosphorylated GhRAF39_1 by GhMPK9 play a leading role in the phosphorylation of GhWRKY40a.

### 2.6 GhMPK9‐GhRAF39_1‐GhWRKY40a Module Play Important Roles in Cotton Resistance to Verticillium wilt

**Figure 6:**
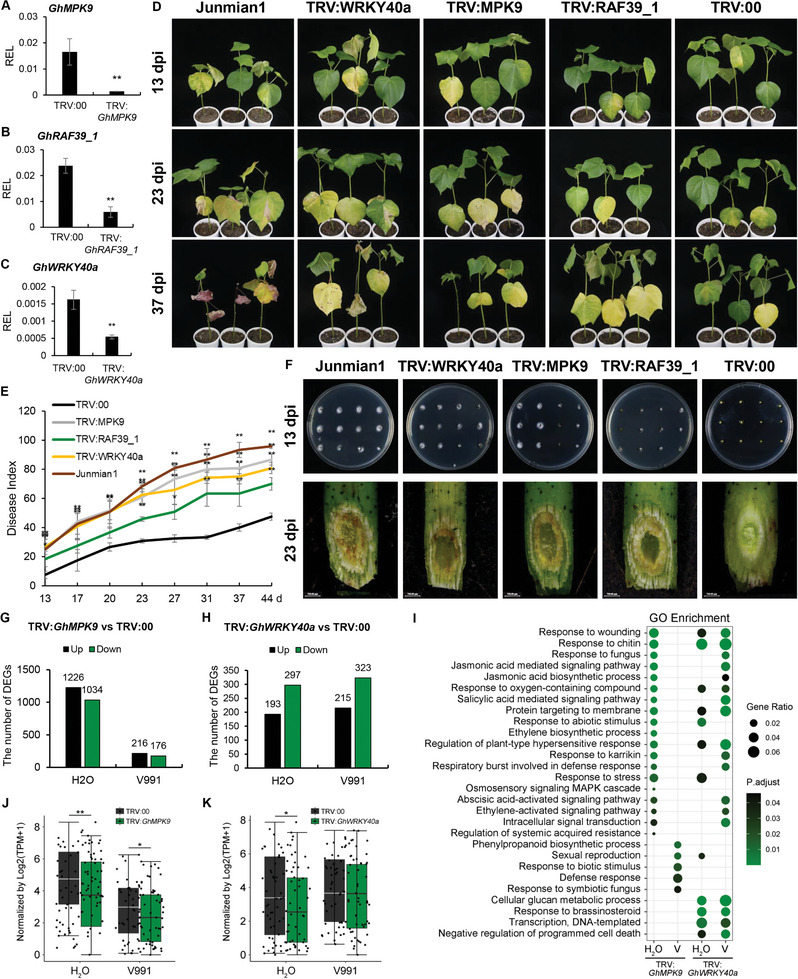
The roles of *GhMPK9, GhRAF39_1*, and *GhWRKY40a* in the resistance to Verticillium wilt in cotton. A–C) The relative expression level (REL) of target genes in silenced plants and control at 14 dpv for *GhMPK9* (A), *GhRAF39_1* (B), and *GhWRKY40a* (C). The values are normalized to those of *Histone3*. D) Phenotypes of cotton seedlings in Junmian1, TRV:*GhMPK9*, TRV:*GhRAF39_1*, TRV:*GhWRKY40a*, and TRV:00 plants from 13 to 37 dpi by V991. E) The disease index of Junmian1, TRV:*GhMPK9*, TRV:*GhRAF39_1*, TRV:*GhWRKY40a* and TRV:00 plants from 13 to 44 dpi. (F) The upper panel displayed the fungal recovery experiments of Junmian1, TRV:*GhMPK9*, TRV:*GhRAF39_1*, TRV:*GhWRKY40a*, and TRV:00 plants at 13 dpi. The lower panel indicated the V991 hyphal growth in their stems at 23 dpi. G,H) The number of up‐ and down‐regulated DEGs between TRV:*GhMPK9* (G) or TRV:*GhWRKY40a* (H) and TRV:00 plants after H_2_O and V991 treatments, respectively. The green column represents the down‐regulated genes and the black column represents the up‐regulated genes. I) GO enrichment analysis of down‐regulated DEGs in TRV:*GhMPK9* and TRV:*GhWRKY40a* plants under normal and V991 conditions. J,K) Boxplots of the expression levels of 51 ethylene‐related genes in TRV:*GhMPK9* plants (J) and TRV:*GhWRKY40a* plants (K) under normal and V991 conditions. Data are presented as means ± SD in (A–C, E, J, and K), *n* = 9 (A–C), *n* = 30 (E), *n* = 51 (J and K), statistical analyses are performed using Student's *t* test: *, *P* < 0.05; **, *P* < 0.01.

To elucidate the putative function of the three genes in disease response, we silenced individually *GhMPK9*, *GhRAF39_1* and *GhWRKY40a* homologs in *G. barbadense* cv. Hai7124, a resistance cotton cultivar, via VIGS strategy. The allotetraploid cottons *G. hirsutum* and *G. barbadense* were derived from a polyploidization event between two diploid ancestors with the A and D genomes. The cultivated *G. hirsutum* is well‐studied in genome research but susceptible to Verticillium wilt. In contrast, *G. barbadense* has stronger resistance to Verticillium wilt. The alignment results showed there were no differences in sequence of *GhMPK9*, *GhRAF39_1*, and *GhWRKY40a* homologs between *G. hirsutum* and *G. barbadense* (Figure S6, Supporting Information). Thus, silencing *GhMPK9, GhRAF39_1*, and *GhWRKY40a* homologs in resistant materials (*G. barbadense*) can exhibit more obvious change in resistance phenotype. The pTRV2 vectors of *GhMPK9_A(D)* (TRV:*GhMPK9*), *GhRAF39_1_A(D)* (TRV:*GhRAF39_1*), *GhWRKY40a_A(D)* (TRV:*GhWRKY40a*) were constructed with TRV:00 and TRV:*GhCLA* as the negative and positive control, respectively. Agrobacterium harboring these constructs were individually infiltrated in cotton cotyledons. When the second leaves of TRV:*GhCLA* seedlings showed albino phenotypes, we collected the second leaves of each *GhMPK9‐*, *GhRAF39_1‐*, and *GhWRKY40a*‐silenced seedlings to verify the target gene silencing efficiency (**Figure** **6**A–C). Afterward, cotton seedlings at three‐true‐leaf stage were root‐inoculated with V991 spore suspensions, with *G. hirsutum* cv. Junmian1, a susceptible cotton cultivar, as control. Compared to TRV:00, the TRV:*GhMPK9*, TRV:*GhRAF39_1* and TRV:*GhWRKY40a* seedlings were more susceptible to Verticillium wilt at 13, 23, and 37 dpi (Figure 6D). The disease index of the TRV:*GhMPK9*, TRV:*GhRAF39_1*, and TRV:*GhWRKY40a* seedlings was significantly higher than that of TRV:00 seedlings from 13 to 44 dpi (Figure 6E). Fungal recovery experiment and vascular tissues of invading *V. dahliae* investigation in cotton stems revealed that the fungal accumulations were higher in TRV:*GhMPK9*, TRV:*GhRAF39_1*, and TRV:*GhWRKY40a* plants than that of TRV:00 plants (Figure 6F). These results revealed that the individual suppression of *GhMPK9*, *GhRAF39_1*, or *GhWRKY40a* could impair the resistance of cotton to Verticillium wilt, implying GhMPK9‐GhRAF39_1‐GhWRKY40a module plays a key role in regulating the response to Verticillium wilt in cotton.

### 2.7 GhWRKY40a Activates *GhERF1b*‐Mediated Pathway to Promote the Downstream Pathogenesis‐Related Genes Expression and Increase Verticillium wilt Resistance

**Figure 7:**
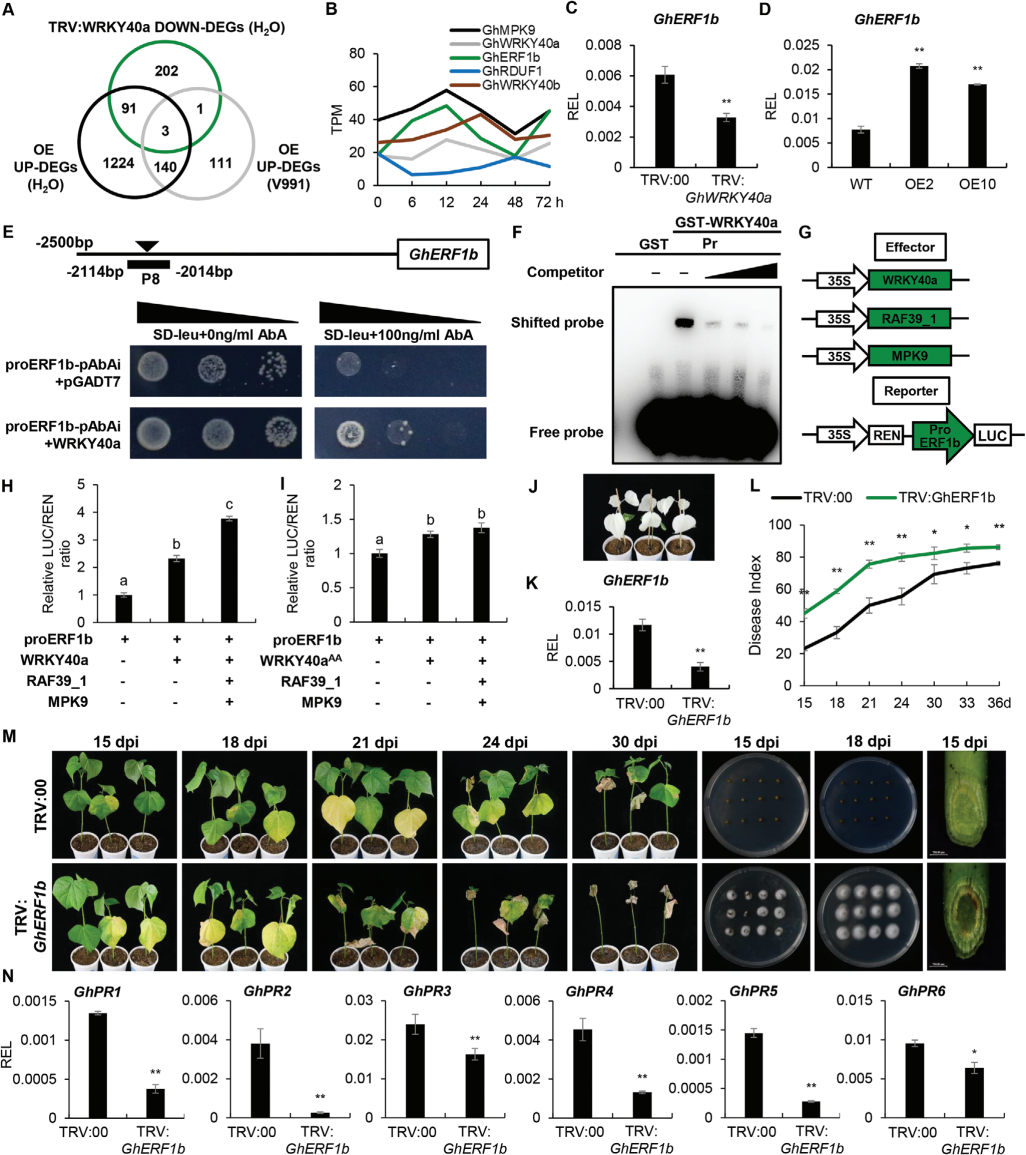
GhMPK9‐GhRAF39_1‐mediated phosphorylation of GhWRKY40a increases its ability to bind the *GhERF1b* promoter and improves resistance to Verticillium wilt in cotton. A) Venn diagram showing the overlap among down‐regulated DEGs in TRV:*GhWRKY40a* and TRV:00 plants under normal conditions and up‐regulated DEGs in both OE2 and OE10 after V991 treatments. *GhERF1b* was found in the overlap. B) The expression patterns of *GhERF1b*, *GhRDUF1*, *GhWRKY40b*, *GhMPK9*, and *GhWRKY40a* after V991 treatments. C,D) The relative expression levels of *GhERF1b* in TRV:*GhWRKY40a* plants (C) and OE lines (D). The values are normalized to those of *Histone3*. E) Yeast one‐hybrid assay showed that GhWRKY40a could bind to P8 of *GhERF1b* promoter. F) Electrophoretic mobility shift assays (EMSA) suggested that GhWRKY40a bind to the *GhERF1b* promoter in vitro. G) Schematic diagrams of the reporter and effector vectors used in the dual‐LUC reporter assay. H) The LUC/REN ratio of promoter of *GhERF1b* with co‐injection of GhMPK9, GhRAF39_1, and GhWRKY40a. I) The LUC/REN ratio of promoter of *GhERF1b* with co‐injection of GhMPK9, GhRAF39_1, and GhWRKY40a^AA^. J) Phenotypes of TRV:*GhCLA* seedlings on 14 dpv. K) The relative expression level of *GhERF1b* in TRV:00 and TRV:*GhERF1b* seedlings on 14 dpv. The values are normalized to those of *Histone3*. L) The disease index of TRV:00 and TRV:*GhERF1b* during 15 to 36 days after V991 inoculation. M) Phenotypes of cotton seedlings in TRV:00 and TRV:*GhERF1b* from 15 to 30 dpi by V991, the fungal recovery experiments of TRV:00 and TRV:*GhERF1b* at 15 and 18 dpi and the V991 hyphal growth in stems of TRV:00 and TRV:*GhERF1b* at 15 dpi. N) The relative expression level of PR genes in TRV:00 and TRV:*GhERF1b* seedlings on 14 dpv. The values are normalized to those of *Histone3*. Data are presented as means ± SD in (C, D, H, I, K, L, and N), *n* = 9 (C, D, H, I, K, and N), *n* = 30 (L), statistical analyses are performed using Student's *t* test: *, *P* < 0.05; **, *P* < 0.01 and one‐way ANOVA test: a/b/c, *P* < 0.05.

To clarify downstream pathways regulated by GhMPK9‐GhRAF39_1‐GhWRKY40a module in modulating the resistance to Verticillium wilt, we performed transcriptome sequencing of root tissues at 12 hpi on TRV:*GhMPK9* and TRV:*GhWRKY40a* seedlings, and compared the DEGs with TRV:00 under normal and V991 conditions. Compared to TRV:00 plants, there were 2260 DEGs in TRV:*GhMPK9* and 490 DEGs in TRV:*GhWRKY40a* under normal conditions as well as 392 DEGs in TRV:*GhMPK9* and 538 DEGs in TRV:*GhWRKY40a* under V991 conditions, respectively (Figure 6G,H; and Data S6, Supporting Information). The GO enrichment analysis indicated the down‐regulated DEGs were mainly involved in response to wounding, chitin and fungus, regulation of plant‐type hypersensitive response, jasmonic acid, salicylic acid, abscisic acid and ethylene‐activated signaling pathway (Figure 6I; and Data S7, Supporting Information). In particular, we observed that many DEGs were related to ethylene signal transduction, in which the transcripts of 51 ethylene‐related genes were significantly lower in TRV:*GhMPK9* or TRV:*GhWRKY40a* than in TRV:00 under both normal and V991 conditions (Figure 6J,K; and Data S8, Supporting Information), indicating that GhMPK9‐GhRAF39_1‐GhWRKY40a promotes the resistance of cotton to Verticillium wilt through regulating the downstream ethylene‐activated signaling pathway. To determine the potential downstream transcription factors, we further compared the DEGs of *GhMPK9* overexpressing transgenic lines and TRV:*GhWRKY40a* plants compared to WT. Only three DEGs were overlapped by comparing down‐regulated DEGs in TRV:*GhWRKY40a* and up‐regulated DEGs in OE under normal and V991 conditions, including *GhERF1b* encoding ethylene responsive element binding factor 1, *GhWRKY40b* encoding WRKY transcription factor, and *GhRDUF1* encoding RING‐type E3 ubiquitin ligases (**Figure** **7**A). Among them, *GhERF1b* had the similar expression pattern with *GhMPK9* and *GhWRKY40a* under V991 condition (Figure 7A,B). The RT‐qPCR analysis confirmed that *GhERF1b* was up‐regulated in *GhMPK9* overexpression lines but down‐regulated in TRV:*GhWRKY40a* plants (Figure 7C,D).

Previous studies showed that the W‐box [(T)TGACC/T] was conserved binding motif for WRKY transcription factors.^[^ ^30^ ^]^ Promoter analysis showed that there were three types of W‐box motifs in the promoter region of *GhERF1b* (Figure S7, Supporting Information). Thus, we divided the promoter of *GhERF1b* into eight segments as baits to perform the yeast one‐hybrid (Y1H) analysis. The results showed that GhWRKY40a could bind to the P8 region of *GhERF1b* promoter (Figure 7E). The electrophoretic mobility shift assay (EMSA) analysis indicated that only GST could not bind to the *GhERF1b* promoter, while GST‐GhWRKY40a could directly bind to the biotin‐labeled probes, which was gradually reduced upon the addition of unlabeled competitive probes (Figure 7F). In addition, we constructed the effector vectors of *GhMPK9*, *GhRAF39_1*, and *GhWRKY40a* and the reporter vector of the *GhERF1b* promoter (Figure 7G). With the help of LUC reporter system, GhWRKY40a could induce the elevated activity of *GhERF1b* reporter, and the co‐expression of GhMPK9, GhRAF39_1, and GhWRKY40a led to the higher activity (Figure 7H). To investigate whether phosphorylation of GhWRKY40a by GhMPK9‐GhRAF39_1 affects its transcriptional activity, we additionally performed dual‐luciferase assays with the phosphorylation mutant of GhWRKY40a (GhWRKY40a^AA^) as the effector. When GhWRKY40a was replaced by GhWRKY40a^AA^, the transcriptional activity of *GhERF1b* reporter was not affected whether joining GhMPK9‐GhRAF39_1 module or not (Figure 7I). These results indicated that GhMPK9‐GhRAF39_1‐mediated phosphorylation of GhWRKY40a significantly increase the promoter activity of *GhERF1b*.

VIGS analysis further confirmed that *GhERF1b* played an important role in cotton response to Verticillium wilt. When the occurrence of leaf albinism in TRV:*GhCLA* seedlings, we detected the expression levels of *GhERF1b* and the silenced plants were inoculated with V991 (Figure 7J,K). The phenotypic observations showed the disease development was apparently more serious, the fungal recovery rate was much faster, the fungal accumulation was greater, and the diseased leaf rate was significantly higher in TRV:*GhERF1b* than that in TRV:00 plants (Figure 7L,M). Also, many pathogenesis‐related (PR) genes were significantly down‐regulated in TRV:*GhERF1b* plants (Figure 7N). Taken together, the GhMPK9‐GhRAF39_1‐mediated phosphorylation of GhWRKY40a activates the transcription of *GhERF1b* and promotes the downstream PR genes expression to increase Verticillium wilt resistance.

### 2.8 GhWRKY40a Inhibits *GhABF2*‐Mediated Pathway to Regulate the Stomatal Opening and Increase Verticillium wilt Resistance

**Figure 8:**
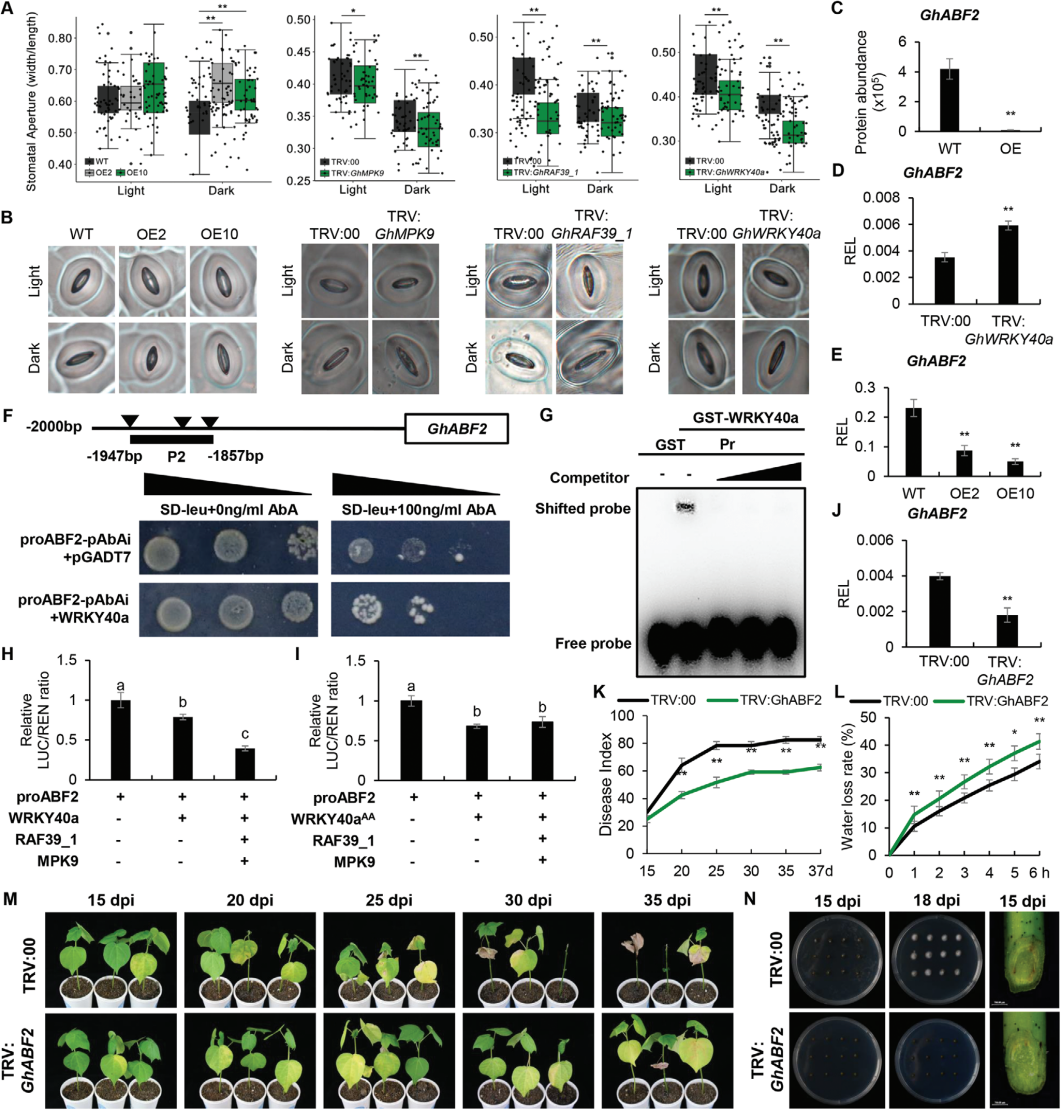
GhMPK9‐GhRAF39_1‐mediated phosphorylation of GhWRKY40a decreases its ability to bind the *GhABF2* promoter whereas improves resistance to Verticillium wilt in cotton. A,B) Boxplots of the stomatal aperture (A) and phenotypes (B) of OE2, OE10, and WT as well as TRV:*GhMPK9*, TRV:*GhRAF39_1*, TRV:*GhWRKY40a*, and TRV:00 plants under light and dark conditions. C) The protein abundance of GhABF2 in WT and OE plants. D,E) The relative expression levels of *GhABF2* in TRV:*GhWRKY40a* plants (D) and OE lines (E). The values are normalized to those of *Histone3*. F) Y1H assay showed that GhWRKY40a could bind to P2 of *GhABF2* promoter. G) EMSA experiments suggested that GhWRKY40a bind to the *GhABF2* promoter in vitro. H,I) GhWRKY40a decreased the transcriptional activity of *GhABF2*. The LUC/REN ratio of promoter of *GhABF2* with co‐injection of GhMPK9, GhRAF39_1, GhWRKY40a, and with co‐injection of GhMPK9, GhRAF39_1, GhWRKY40a^AA^, respectively. J) The relative expression level of *GhABF2* in TRV:00 and TRV:*GhABF2* seedlings on 14 dpv. The values represent means ± SD, *n* = 9, and normalized to those of *Histone3*. K) The disease index of TRV:00 and TRV:*GhABF2* plants during 10 to 37 days after V991 inoculation. Values represent means ± SD, *n* = 30. L) The water loss rate of TRV:00 and TRV:*GhABF2* plant leaves from 0 to 6 h. Values represent means ± SD, *n* = 30. M) Identification of disease resistance of cotton seedlings between the TRV:00 and TRV:*GhABF2* plants from 15 to 35 dpi by V991. N) The fungal recovery experiments of TRV:00 and TRV:*GhABF2* at 15 and 18 dpi and the V991 hyphal growth in stems of TRV:00 and TRV:*GhABF2* at 15 dpi. Data are presented as means ± SD in (A, C, D, E, and H–L), *n* = 50 (A), *n* = 3 (C), *n* = 9 (D, E, and H–J), *n* = 30 (K), *n* = 12 (L), statistical analyses are performed using Student's *t* test: *, *P* < 0.05; **, *P* < 0.01 and one‐way ANOVA test: a/b/c, *P* < 0.05.

While analyzing the transcriptome data of *GhMPK9* overexpression lines, we also noticed that overexpressing *GhMPK9* could induce changes in the regulation of stomatal complex development/patterning (Figure 2J). Stomata determines the internal temperature and humidity of plants by mediating gas exchange and water vapor, which can directly influence the infecting extent of *V. dahliae*.^[^ ^31^ ^]^ We speculate that overexpression of *GhMPK9* probably affect the resistance of cotton by regulating stomatal opening and closure. We first investigated the phenotype of stomata on the leaf epidermis of OEs and WT plants. For light‐induced stomatal opening measurements, there were no significant differences in stomatal aperture between OE lines and WT plants, but there were obvious obstacles to stomatal opening in TRV:*GhMPK9*, TRV:*GhRAF39_1*, and TRV:*GhWRKY40a* plants, respectively (**Figure** **8**A,B). For dark‐treated stomatal closure assays, the stomatal apertures of OE lines were more than those of WT plants, while the stomatal aperture of TRV:*GhMPK9*, TRV:*GhRAF39_1*, and TRV:*GhWRKY40a* plants were all significantly smaller than TRV:00 plants (Figure 8A,B). These results suggested that *GhMPK9*, *GhRAF39_1*, or *GhWRKY40a* participate in stomatal opening and closure in cotton.

To elucidate the mechanism of *GhMPK9*‐mediated stomatal regulation, we further analyzed phosphor‐proteomic results and found the protein abundance of GhABF2 was significantly reduced in the *GhMPK9*‐overexpressing plants (Figure 8C). Recent research reported that AtABF2 could bind to the abscisic acid as a leucine zipper transcription factor, which participated in the regulation of stomatal aperture by directly regulating the expression of *AtTPPI*.^[^ ^32^ ^]^ We found that the expression levels of *GhABF2* was drastically increasing in TRV:*GhWRKY40a* plants, but significantly decreasing in *GhMPK9* overexpressing lines (Figure 8D,E). Furthermore, the W‐boxes binding elements of GhWRKY40a were also analyzed in the *GhABF2* promoter. We divided the promoter of *GhABF2* into two segments as baits to perform the Y1H analysis (Figure S8, Supporting Information). The result demonstrated that GhWRKY40a could directly bind to the P2 region of *GhABF2* promoter (Figure 8F). The EMSA experiments showed GhWRKY40a could directly bind to the P2 region, and this binding was gradually reduced by the addition of non‐radiolabeled competitive oligo probes (Figure 8G). The LUC reporter system assays indicated the co‐injection of GhMPK9, GhRAF39_1 and GhWRKY40a significantly reduced the transcriptional activity of *GhABF2* (Figure 8H), while GhMPK9 and GhRAF39_1 did not affect the transcriptional inhibition of *GhABF2* by GhWRKY40a^AA^ (Figure 8I). Moreover, silencing *GhABF2* confers the lower rate of diseased leaves, the higher rate of water loss, and the less fungal accumulation in stems, indicating *GhABF2* functions as a negative regulator in the response of cotton to Verticillium wilt (Figure 8J–N). Thus, the GhMPK9‐GhRAF39_1‐mediated phosphorylation of GhWRKY40a inhibits the transcription of *GhABF2*, and induces stomatal opening and reduces internal humidity of plants, thereby enhancing cotton resistance to Verticillium wilt.

## 3 Discussion

The mitogen‐activated protein kinase cascade is a highly conserved class of pathways in plants that play a crucial role in plant immunity. Activation of the MAPK cascade is one of the earliest events in response to pathogen invasion.^[^ ^33^ ^]^ In this study, we found a novel molecular module of GhMPK9‐GhRAF39_1‐GhWRKY40a, which GhMPK9 could not phosphorylate directly GhWRKY40a, GhRAF39_1 as a key protein kinase mediated the phosphorylation of GhWRKY40a by GhMPK9, and the phosphorylated GhWRKY40a regulated the downstream *GhERF1b* and *GhABF2* expression, thereby enhancing the resistance to Verticillium wilt. Our findings revealed a new role of Raf‐like kinase, enriched the types of MAPK substrates in plants, and elucidated the dual functions of GhWRKY40a in increasing the resistance to Verticillium wilt in cotton.

### 3.1 GhMPK9 Plays an Important Role in Cotton Growth and Development and Immune Response

The MAPK gene family is widely involved in signal transduction processes of plant growth, development, and stress response. Among them, MAPK3 is the most extensively studied protein kinase in plants, which mainly functions in ovule development and disease response. In *Arabidopsis thaliana*, *AtMPK3* has redundant functions with *AtMPK6*. Previous studies reported single loss‐of‐function mutants of *mpk3* or *mpk6* usually show normal phenotypes but the *mpk3*/*mpk6* double mutants is embryo lethal.^[^ ^8^, ^19^, ^34^ ^]^ In cotton, there were four pairs of homologous genes of *AtMPK3/6*, including *GhMPK3*, *GhMPK6*, *GhMPK9*, and *GhMPK13* (Figure 1A). Of them, *GhMPK9* and *GhMPK13* were highly expressed in floral organs (Figure 1B). Our studies showed that suppressing *GhMPK9* could cause completely female and male sterility of floral organs, different from the redundant function of homologous genes in *Arabidopsis thaliana*, indicating *GhMPK9* plays an independent role in sexual reproduction in cotton (Figure S2A–D, Supporting Information).

In the pattern‐triggered immunity or effector‐triggered immunity signaling pathway, *AtMPK3/6* was intensely but transiently activated by both flg22 and elf18 and played a critical role in the regulation of pathogen‐induced ethylene biosynthesis and camalexin biosynthesis.^[^ ^9^, ^10^, ^11^, ^35^ ^]^ MPK3/6 have been implicated in the activation of various immune responses and their inactivation leads to compromised defense against pathogens.^[^ ^36^ ^]^ In this study, we observed *GhMPK9* was rapidly induced by *V. dahliae* in cotton and then it gradually dropped down (Figure 1D). We infer the regulation mechanism of GhMPK9 is working, which can be supported by obvious variations in the amount of DEGs between OE2 and OE10 based on the results of RNA‐seq (Figure 2F,H). *GhMPK9* has higher transcription levels in OE2, which may activate excessive immune responses. Conversely, the moderate expression of *GhMPK9* in OE10 can activate both immune responses and diverse cellular responses (Data S2, Supporting Information). These results reflect the importance of *GhMPK9* precisely controlling the growth and development and immune defense in cotton.

### 3.2 GhRAF39_1 as Helper Functions in Phosphorylation of GhWRKY40a by GhMPK9 in Immune Signaling

Identifying MAPK substrates is an efficient way to help us comprehend the potential mechanism of MAPK signaling. To date, many transcription factors, enzymes, structural proteins, and kinases are identified as phosphorylated substrates of MAPKs through a phospho‐proteomic analyses.^[^ ^2^, ^37^ ^]^ Most studies in plants have shown that the activation of MPK3/MPK6 leads to the phosphorylation of its downstream transcription factors in the immune signaling pathway, such as WRKY33, ACS2, ACS6, ERF6, ERF104 in *Arabidopsis thaliana*, WRKY53 in rice and WRKY17 in apple.^[^ ^9^, ^10^, ^12^, ^35^, ^38^ ^]^ In our studies, GhMPK9 could physically interact with transcription factor GhWRKY40a, while it could not phosphorylate directly GhWRKY40a, implying that there may exist an unknown protein as mediator. Phospho‐proteomic analysis further identified that a Raf‐like kinase GhRAF39_1 as MAPK substrate mediated the phosphorylation of GhWRKY40a by GhMPK9. In the plant MAPK cascade, there are two groups of MAPKKKs, MEKK1‐like and Raf‐like MAPKKKs, in which typical MAPKKKs can positively regulate the downstream MAPKK‐MAPK module, including MEKK1, MAPKKK3, MAPKKK5 and YDA.^[^ ^3^, ^19^, ^39^ ^]^ As for Raf‐like members, there are few evidences to support they can activate downstream MAPKK‐MAPK modules, as bona fide MAPKKKs.^[^ ^40^ ^]^ Surprisingly, MPK6 also phosphorylates MAPKKK5 to improve MPK3/6 activity and resistance to disease, suggesting a positive feedback mechanism.^[^ ^41^ ^]^ Conversely, OsEDR1 as a Raf‐like kinase was found to inhibit the activity of OsMPKK10.2 via physical interaction in rice and it could also be phosphorylated by OsMPK6 to disinhibit OsMKK10.2, leading to the enhanced resistance to *Xoc*.^[^ ^6^ ^]^ Our studies indicate that GhRAF39_1 functions as the downstream substrate of GhMPK9 rather than its upstream kinase, suggesting that GhRAF39_1 might not be a traditional “MAPKKK” in MAPK‐mediated immune signaling. We proved GhRAF39_1 could physically interact with GhWRKY40a in vitro and in vivo, but only when it was phosphorylated by GhMPK9, the phosphorylated GhRAF39_1 could further activate GhWRKY40a phosphorylation (Figures 3, 4, 5). In addition, we also proved that GhMPK9‐GhRAF39_1‐GhWRKY40a through sequential phosphorylation regulated the expression of downstream transcription factors to increase the resistance to Verticillium wilt in cotton (Figure 6). Thus, this study reports a novel role of Raf‐like MAPKKK GhRAF39_1, which functions as middle regulators to link MAPK and its downstream transcription factors in plant immunity.

### 3.3 GhMPK9‐GhRAF39_1‐GhWRKY40a Positively Regulates the Resistance to Verticillium wilt via *GhERF1b*‐Mediated Ethylene Signal Transduction in Cotton

Ethylene plays various roles in plant disease resistance, such as increasing resistance of plants, hastening the aging and leaf‐shedding processes of plants.^[^ ^42^ ^]^ In addition, ethylene may also affect other disease resistance mechanisms, thus increasing the susceptibility of plants to diseases.^[^ ^43^ ^]^ When *G. barbadense* was inoculated by *V. dahliae*, aminoacyl‐cyclopropane oxidase (ACO) was quickly up‐regulated.^[^ ^44^ ^]^ After treating with exogenous ethylene and *V. dahliae*, the expression of ACO1 was significantly upregulated, indicating that ethylene biosynthesis may be related to the resistance of cotton at the early stages of Verticillium wilt infection.^[^ ^45^ ^]^ Ethylene response factor (ERF) functions in plant disease resistance as a member of the AP2/ERF transcription factor family downstream of ethylene signaling pathway. *GbERF1*‐like ethylene response‐related factor enhances the resistance to *V. dahliae* through lignin biosynthesis.^[^ ^46^ ^]^ *AtERF6* plays important roles in regulating plant defense against fungal pathogens, as an ethylene‐independent transcription factor downstream of the *AtMPK3/6* cascade.^[^ ^12b^ ^]^ Overexpression of *AtERF1* enhances resistance through the increased expression of *AtPDF1.2* and *AtPR3*.^[^ ^42^, ^47^ ^]^ AtMPK3/6 can phosphorylate AtERF1 to increase its transactivation activity, and AtERF1 interacts with AtWRKY33 to form transcriptional complexes, thereby mediating the synergy of ethylene/JA and MPK3/MPK6 signaling pathways to induce camalexin biosynthesis.^[^ ^48^ ^]^ In this study, we found that GhMPK9‐GhRAF39_1‐GhWRKY40a module promoted the *GhERF1b* transcription to increase the resistance to Verticillium wilt. *GhERF1b* is a homolog of *AtERF1*. Silencing *GbERF1* increases susceptibility to *V. dahliae*, whereas overexpressing *GbERF1* enhances the resistance through activating lignin synthetic genes.^[^ ^46^ ^]^ We proved that GhMPK9‐GhRAF39_1 module could strengthen the binding capacity of GhWRKY40a to the *GhERF1b* promoter and silencing *GhERF1b* could decrease the expression of PR genes and thereby increase the susceptibility to *V. dahliae* in cotton (Figure 7D–M). Our study elucidates a regulatory mechanism downstream of GhMPK9‐GhRAF39_1‐GhWRKY40a, that promotes the transcription of *GhERF1b* and activates disease‐resistant genes to enhance the cotton resistance to Verticillium wilt.

### 3.4 GhMPK9‐GhRAF39_1‐GhWRKY40a Negatively Regulates the Resistance to Verticillium wilt via *GhABF2*‐Mediated the Stomatal Regulation in Cotton

Stomata play a critical role in gas exchange and water evaporate of plants.^[^ ^49^ ^]^ Stomatal closure prevents water loss and limits pathogen entry, also reduces photosynthesis and transpiration and creates high temperature and humidity conditions that promote colonization by pathogens.^[^ ^50^ ^]^ Recently, it has been found that the opening of stomata prevents the pathogen from colonizing the plant thereby increasing disease resistance.^[^ ^31^ ^]^ In our studies, *GhMPK9*‐overexpression transgenic plants showed the enhanced resistance to Verticillium wilt and kept stomatal opening under dark conditions. Conversely, TRV:*GhMPK9*, TRV:*GhRAF39_1* and TRV:*GhWRKY40a* plants showed the decreased resistance to Verticillium wilt and maintained stomatal closure whatever under light and dark conditions (Figure 8A,B). Phosphor‐proteome analysis found a significant reduction in protein abundance of GhABF2, a key bZIP transcription factor in abscisic acid (ABA) response signaling pathway, in *GhMPK9*‐overexpression transgenic plants (Figure 8C), indicating that *GhMPK9* play a key role in regulating the stomatal opening and closure. In *Arabidopsis thaliana*, *AtABF2* overexpression is associated with increased tolerance to drought, high salt, high temperature and oxidative stress, and reduced transpiration rates. Its strong promoter activity in guard cells were also found to have an active role in stomatal closure.^[^ ^51^ ^]^ Recently, ABA response element binding factors ABF1, ABF2 and ABF4 enhance drought tolerance by directly regulating AtTPPI (trehalose‐6‐phosphate phosphatase) expression to reduce stomatal opening and improve root architecture.^[^ ^32^ ^]^ ABF2 binds directly to the TPPE (member of TPPs) promoter to activate its expression and control stomatal closure and root growth.^[^ ^52^ ^]^ We found that GhMPK9‐GhRAF39_1‐ GhWRKY40a could inhibit the transcriptional activity of the *GhABF2* promoter and silencing *GhABF2* could increase the loss of moisture in vivo, thereby improving the resistance to *V. dahliae* in cotton (Figure 8D–N). Thus, our study revealed another regulatory mechanism mediated by GhMPK9‐GhRAF39_1‐GhWRKY40a, that prevents stomatal closure to reduce the internal temperature and humidity through suppressing the transcription of *GhABF2* in cotton, so that it can inhibit the colonization of *V. dahliae*.

**Figure 9:**
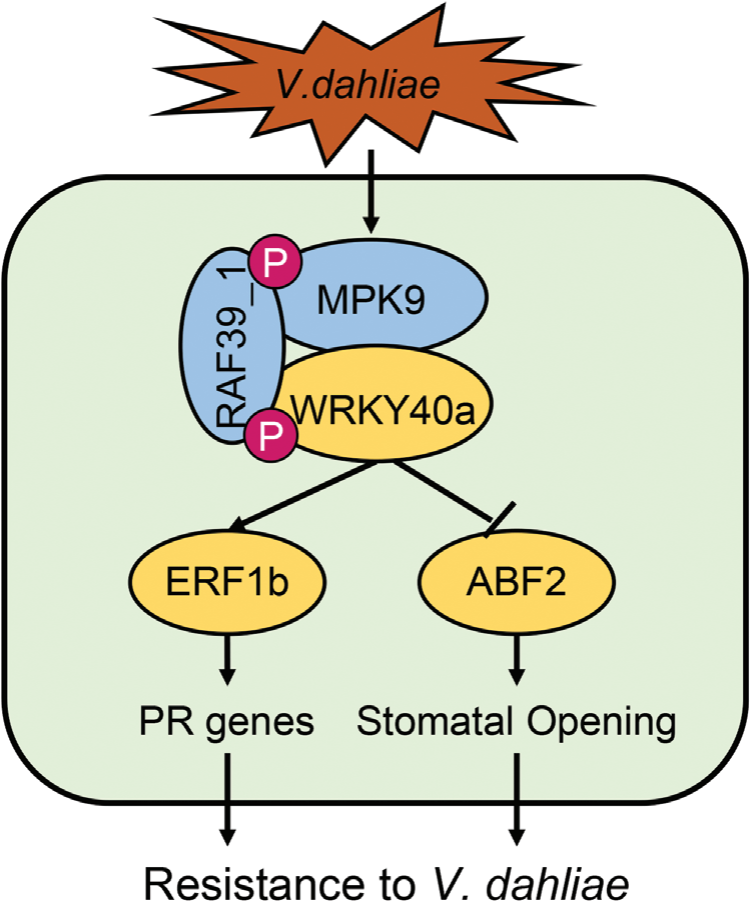
A model of GhMPK9‐GhRAF39_1‐GhWRKY40a module for increasing the resistance to *Verticillium dahliae* in cotton. When *V. dahliae* attacks, GhMPK9 is activated by unknown kinases, GhMPK9‐GhRAF39_1‐GhWRKY40a in sequential phosphorylation activates GhWRKY40a. The phosphorylated GhWRKY40a had the dual functions, which enhances the transcriptional activity of *GhERF1b* to promote the expression levels of PR genes, and suppresses the transcriptional activity of *GhABF2* to regulate the leaf stomata opening causing the loss of leaf moisture. Both the expression of PRs and the leaf stomata opening lead to the inhibition of fungal colonization, thereby improving the resistance to *V. dahliae* in cotton. The blue ellipse represents the protein kinases. The yellow ellipse represents the transcription factors. Arrows indicate activation, bars indicate inhibition, and dashed lines indicate postulated regulation.

Based on these, we propose a novel GhMPK9‐GhRAF39_1‐GhWRKY40a module functions as a positive regulator for increasing the resistance to *V. dahliae* in cotton. When *V. dahliae* infects, *GhMPK9* is activated by unknown kinases, GhMPK9 phosphorylated GhRAF39_1, and the activated GhRAF39_1 phosphorylated GhWRKY40a. The activated GhWRKY40a played the dual functions, which enhances the transcriptional activity of *GhERF1b* to increase the expression levels of PR genes, and suppresses the transcriptional activity of *GhABF2* to regulate the leaf stomata opening and result in the loss of leaf moisture. Both the expression of PRs and the leaf stomata opening lead to the inhibition of fungal colonization, thereby improving the resistance to *V. dahliae* in cotton (**Figure** **9**).

## 4 Experimental Section

### Plant Materials, Treatments, and Sample Collection

*Gossypium hirsutum* L. acc. TM‐1 and *G. hirsutum* L. cv. Junmian1 showing susceptibility to *V. dahliae*, *G. barbadense* L. cv. Hai7124 showing resistance to *V. dahliae*, and *GhMPK9* transgenic cotton plants generated from *G. hirsutum* acc. W0 as receptor, were grown in the greenhouse maintained at a constant temperature of 25 °C under long‐day conditions with a 16/8‐h light/dark photoperiod and a relative humidity of 60% for various use. *Nicotiana benthamiana* plants were grown in the greenhouse with a 23 °C /21 °C (16‐h light/8‐h dark) cycle for subcellular localization and interaction assays. For *V. dahliae* inoculation, cotton seedlings were grown until reaching the two‐leaf stage and then treated with 30 mL per plant of 1 × 107 spore mL^−1^ V991 for once using the root‐dip method (Sink and Grey, 1999). The roots of the control and treated plants were harvested at each time point. All samples were immediately put in liquid nitrogen and then store at −80 °C. Experiments were maintained at a constant temperature of 25 °C under long‐day conditions with a 16/8‐h light/dark photoperiod and a relative humidity of 60%.

### RNA Isolation and RT‐qPCR Analysis

CTAB‐acidic phenolic method was used to isolate cotton total RNA,^[^ ^53^ ^]^ and the HiScript III RT SuperMix (+ gDNA wiper) (Vazyme, Inc., China) was used to reverse‐transcribe the total RNA into cDNAs according to the manufacturer's instructions. Reverse transcription quantitative PCR (RT‐qPCR) was performed on a CFX96 Touch Real‐Time PCR detection system (Bio‐Rad, CA, USA) using AceQ SYBR Green Master (Vazyme, Inc., China). Gene‐specific primers for RT‐qPCR analysis were designed using Beacon Designer 7.0 software (Premier Biosoft International, CA, USA). Cotton Histone 3 (GenBank ID: AF024716) was used as reference gene.^[^ ^54^ ^]^ All primer information is listed in Table S2 (Supporting Information). The experiments were performed using three biological replicates for each independent sample and three technical replicates for each biological replicate. The gene relative expression levels were calculated following Livak and Schmittgen method.^[^ ^55^ ^]^

### VIGS Assays

To perform VIGS experiment, the specific fragments in the coding sequences of *GhMPK9*, *GhRAF39_1*, *GhWRKY40a*, *GhERF1b*, and *GhABF2* were amplified from the cDNA using the PCR detection system (Bio‐Rad, CA, USA). Then, fragments were separately cloned into the TRV:00 plasmid at the *Eco*RI and *Bam*HI sites using the In‐Fusion HD Cloning Kit (Clontech) according to the protocol provided by the manufacturer. The recombinant plasmids were transformed into *A. tumefaciens* strain GV3101. TRV vectors were injected into two fully expanded cotyledons of *G. barbadense* cv. H7124 plants as described previously.^[^ ^56^ ^]^ The VIGS‐infected seedlings were cultured in a 25 °C controlled growth chamber with a 16/8‐h light/dark photoperiod. At least 30 plants were inoculated with each construct. The TRV:*GhCLA* construct served as a positive marker for evaluating VIGS efficiency.^[^ ^57^ ^]^ After 2 weeks injection, the expression levels of the target gene were examined, and the successfully silenced plants were selected for subsequent experiments. The primers used in this study are listed in Table S2 (Supporting Information).

### Plasmid Construction and Genetic Transformation

The open reading frame (ORF) of *GhMPK9*, *GhRAF39_1*, *GhWRKY40a*, *GhERF1b*, and *GhABF2* were amplified from the cDNA of TM‐1 using the PCR detection system (Bio‐Rad, CA, USA). The ORF of *GhMPK9* fused into the vector pCAMBIA2301 at the *Xba*I and *Sma*I restriction sites to generate the overexpression vector. The 318 bp of *GhMPK9* was inserted into pART27 to generate the *GhMPK9*‐RNAi vector. The *GhMPK9* overexpression and RNAi vectors were used to transform G. *hirsutum* acc. W0 by *A. tumefaciens* (strain LBA4404). The *A. tumefaciens*‐mediated cotton transformation was performed following the method described previously.^[^ ^58^ ^]^ The primers used in this study are listed in Table S2 (Supporting Information).

### Subcellular Localization Analysis

The ORFs of *GhMPK9*, *GhRAF39_1*, *GhWRKY40a* without stop codon fused into upstream of enhanced green fluorescent protein (eGFP), the pBIN‐35S::GhMPK9‐eGFP4, pBIN‐35S::GhRAF39_1‐eGFP4, and pBIN‐35S::GhWRKY40a‐eGFP4 vectors with *Kpn*I and *Bam*HI restriction enzyme sites were generated. pBIN‐35S::eGFP4, pBIN‐35S::GhMPK9‐eGFP4, and pBIN‐35S::GhRAF39_1‐eGFP4 were transiently expressed in *N. benthamiana* leaves following the infiltration method.^[^ ^59^ ^]^ The pBIN‐35S::eGFP4 or pBIN‐35S::GhWRKY40a‐eGFP4 was transiently co‐expressed with nuclear‐localized AtHTB2‐mCherry.^[^ ^60^ ^]^ After 3d infiltration, fluorescence in the tobacco epidermal cell was observed using a confocal laser scanning microscope at the specific excitation and emission wavelengths (eGFP, 488 and 507 nm; mCherry, 587 and 610 nm) (LSM 780, Zeiss, Germany).

### Yeast‐Two‐Hybrid Assays

The ORFs of *GhMPK9*, *GhRAF39_1* fused into the vector pGBKT7 to generate bait plasmid, and the ORFs of *GhWRKY40a*, *GhRAF39_1* fused into the vector pGADT7 to generate prey plasmid. The experiment was performed using the Matchmaker Gold Yeast Two‐Hybrid System (Clontech, Japan) according to the manufacturer's recommended protocol. The appropriate combinations of these recombinant plasmids were co‐transformed into *Saccharomyces cerevisiae* strain Y2H and plated on without Leu and Trp (SD/‐Leu/‐Trp). To detect interactions, transformants were plated on medium supplemented with 40 mg L^−1^ X‐α‐Gal lacking Leu, Trp, His, and Ade (SD/‐Leu/‐Trp/‐His/‐Ade QDO).

### Protein Expression and Purification

Full‐length *GhMPK9* and *GhRAF39_1* were inserted into *pGEX‐4T‐2* vector with *Bam*HI and *Eco*RI restriction enzyme sites to generate GST‐GhMPK9 and GST‐GhRAF39_1. In order to generate His‐GhMPK9 and His‐GhWRKY40a vectors, DNA fragments of *GhMPK9* and *GhWRKY40a* were amplified by a PCR reaction, respectively, followed by cloned into the *pET30a* vector digested with *Bam*HI and *Eco*RI. The open‐reading frame of *GhMPK9* was cloned into the *Eco*RI and *Bam*HI sites of *pMAL‐c2X* to generate MBP‐GhMPK9. Each plasmid was transformed into *E. coli* BL21 (DE3) and protein expression was induced by addition of 0.5 mm IPTG followed by incubation at 16 °C for 16 h. The MBP‐GhMPK9 protein was purified with MBP Bind Resin. The GST‐GhMPK9 and GST‐GhRAF39_1 protein was purified using the GST magnetic beads (Solarbio Science & Technology Co., Ltd, Beijing China). The His‐GhMPK9 and His‐GhWRKY40a proteins were purified using the His magnetic beads (Solarbio Science & Technology Co., Ltd, Beijing China).

### Glutathione S‐Transferase (GST) Pull‐Down Assays

For pull‐down assay, purified protein of GST or GST‐GhRAF39_1 or GST‐GhMPK9 immobilized on the GST magnetic beads (Solarbio Science & Technology Co., Ltd, Beijing China) were incubated with His‐GhMPK9 or His‐GhWRKY40a in PBS at 4 °C overnight. The proteins were eluted from beads after washing them for three times using washing buffer. The proteins were boiled in 5×SDS loading buffer and separated by 10% SDS‐PAGE. The His antibody (GenScript Biotech, Nanjing, China) and GST antibody (Solarbio Science & Technology Co., Ltd, Beijing China) were used to detect the proteins.

### Bimolecular Fluorescence Complementation (BiFC) Assay

For BiFC analysis, *GhRAF39_1* and *GhMPK9* were cloned into the pSPYNE173 vector, and *GhMPK9*, *GhWRKY40a* were cloned into the pSPYCE vector. The recombinant vectors were transformed into *Agrobacterium tumefaciens* GV3101 and co‐expressed in *N. benthamiana* leaves. The infiltrated *N. benthamiana* plants for 16 h were incubated in the dark and then under normal growth conditions at 25 °C for 2 days. The YFP fluorescence was visualized under a confocal laser‐scanning microscope (LSM780, Carl Zeiss, Jena, Germany).

### Luciferase Complementation Imaging Assays

Full‐length *GhRAF39_1* and *GhWRKY40a* were inserted into the pCAMBIA1300‐nLUC vectors, full‐length *GhRAF39_1*, and *GhMPK9* were inserted into the pCAMBIA1300‐cLUC vectors. The recombinant vectors were transformed into *A. tumefaciens* strain GV3101. *N. benthamiana* leaves were injected using the infiltration method. The LUC signal was observed at 72 h post‐transfection using a Tanon 5200 multi‐chemiluminescent imaging system.

### In Vitro Phosphorylation Assay

The purified MBP‐GhMPK9 recombinant protein and purified GST‐GhRAF39_1 recombinant protein or the purified MBP‐GhMPK9 recombinant protein and purified His‐GhWRKY40a recombinant protein were incubated with kinase reaction buffer (25 mm Tris‐HCl [pH 7.5], 12 mm MnCl_2_, 1 mm DTT, 10 µCi [γ−32P]‐ATP) at 30 °C for 30 min for isotope phosphorylation assays in vitro. The reactions were stopped by adding 5 × SDS loading buffer and boiled for 5 min in a metal bath. Proteins were separated by 10% SDS‐PAGE. The protein gels were washed twice with trichloroacetic acid (TCA) solution (5% TCA, 1% NaPPi) for 10 min each, wrapped in dialysis glassine paper and allowed to dry naturally. Phosphorylated proteins were detected by autoradiography using a Typhoon Trio multifunctional laser scanning imager (General Electric, Fairfield, CT, USA). The protein inputs were detected by Coomassie brilliant blue (CBB) staining. For western blot analysis, the purified GST‐GhRAF39_1 recombinant protein and purified His‐GhWRKY40a recombinant protein or the purified MBP‐GhMPK9 recombinant protein and purified GST‐GhRAF39_1 and purified His‐GhWRKY40a were incubated with kinase reaction buffer (25 mm Tris‐HCl [pH 7.5], 10 mm MnCl_2_, 1 mm DTT, 200 µm ATP) at 30 °C for 30 min. The reactions were stopped by adding 5 × SDS loading buffer and boiled for 5 min. Samples were separated by 10% SDS‐PAGE followed by immunoblotting with biotin‐pendant Zn^2+^‐Phos‐tag (BTL‐111) according to the manufacturer's instructions (Western blot analysis of phosphorylated proteins‐chemiluminescent detection using biotinylated Phos‐tag; Nard, https://www.nard.co.jp).

### Yeast One‐Hybrid (Y1H) Assays

Yeast‐one‐hybrid (Y1H) assays were conducted using the Matchmaker Gold Yeast One‐Hybrid System (Clontech Laboratories, Inc.). The W‐box sequences of the *GhERF1b* were divided into 8 segments(E1‐E8), the W‐box sequences of the *GhABF2* promoter were divided into 2 segments (A1, A2), and these sequences were inserted into the pAbAi vector. These plasmids were linearized and transformed into Y1H Gold strain. The CDSs of *GhWRKY40a* was inserted into the pGADT7 vector. pGADT7‐*GhWRKY40a* and empty pGADT7 were transformed respectively into yeast strain pro*GhERF1b*::pAbAi or pro*GhABF2*::pAbAi, after which the transformed strains were cultured on basal media (SD media) lacking Leu (SD/−Leu) with or without 100 ng mL^−1^ aureobasidin A (AbA). The primers used in this study are listed in Table S2 (Supporting Information).

### Luciferase (LUC) Reporter System Assays

The CDSs of *GhMPK9*, *GhRAF39_1*, and *GhWRKY40a* subsequently were cloned into the effector (35S‐transcription factor) vectors to generate 35S::*GhMPK9*, 35S:: *GhRAF39_1*, and 35S:: *GhWRKY40a*. The promoters of *GhERF1b* and *GhABF2* were cloned into the reporter (SNBE‐mini35S‐luciferase) vectors to generate pro*GhERF1b*::*LUC* and pro*GhABF2*::*LUC* recombinant plasmids. Empty effector (35S‐transcription factor) vector used as a negative control. The reporter and effector vectors were transformed into *A. tumefaciens* strain *GV3101* and injected into *N. benthamiana* leaves. The activities of luciferase were detected using the Dual Luciferase Reporter Assay kits (Vazyme Inc., Nanjing, Jiangsu, China) and recorded using a Multi‐Mode Microplate Reader (SpectraMax iD5).

### Electrophoretic Mobility Shift Assays (EMSA)

Electrophoretic mobility shift assays (EMSAs) were performed using a Chemiluminescent EMSA Kit (Beyotime, Shanghai, China). The ORF of *GhWRKY40a* was constructed into the vector *pGEX‐4T‐2*, and the GST‐tagged protein was expressed *Escherichia coli* BL21 (DE3) and induced by the addition of 0.5 mm IPTG followed by incubation at 16 °C for 16 h. The GST‐GhWRKY40a protein were purified using the GST magnetic beads (Solarbio Science & Technology Co., Ltd, Beijing China). GST protein was used as a negative control. Biotin was used to label the 50‐bp promoter fragments of *GhERF1b* and *GhABF2* containing the native W‐box and used as the detection probes. The same unlabeled fragments were used as the competitor probes competed with the labelled probes. Protein inputs were detected using an anti‐GST antibody (GenScript Biotech, Nanjing, China). Primers used are listed in Table S2 (Supporting Information).

### Measurement of Stomatal Aperture

For stomatal aperture measurements, fully expanded young leaves of 3‐week‐old cotton plants were treated. For assays of stomatal closure, leaves were incubated in the stomatal closure buffer (20 mm KCl, 1 mm CaCl_2_, 5 mm MES‐KOH, pH 6.15) for 2.5 h in the light, then 2.5 h in the dark. For light‐induced stomatal opening, leaves were incubated in the stomatal opening buffer (30 mm KCl and 10 mm MES‐KOH, pH 6.5) for 2 h in the dark, followed by 2.5 h in the light.^[^ ^61^ ^]^ Stomatal apertures were photographed immediately on a microscope slight with a light microscope (Olympus BX53). After image acquisition, the width of stomatal apertures was measured with the open access software Image J (Version 1.37).

### Transcriptome Analysis

For transcriptome analysis, cotton seedlings were grown in soil under normal watering conditions. After three weeks, root samples from each group at 12 h after treatments were collected. Each group had three biological replicates. Each biological replicate contained three individual plants. All samples were collected for each treatment group at each time point. All samples were immediately put in liquid nitrogen and then store at −80 °C. The high‐throughput sequencing service was provided by Novogene Co, Ltd. (Beijing, China) with the NovaSeq 6000 sequencing system (Illumina, CA, USA). The quality of RNA‐seq raw reads were controlled by Cutadapt software, and then the clean reads were mapped to the cotton reference genome *Gossypium hirsutum* “TM‐1′ HAU_v1 and *Gossypium barbadense* ‘Hai7124” HAU_v2 using Hisat2.^[^ ^62^ ^]^ HTSeq was used to count the filtered reads of all cotton annotated genes,^[^ ^63^ ^]^ and the R package edgeR was used for differential expression analysis.^[^ ^64^ ^]^ Gene expression values were normalized by transcripts per kilobase of exon model per million mapped reads (TPM) using String Tie.^[^ ^65^ ^]^ The R package WGCNA was used for gene co‐expression network analysis.^[^ ^66^ ^]^ The R package ClusterProfiler was used for GO enrichment analysis.^[^ ^67^ ^]^ For the convenience of data comparison between two cotton accessions, the gene ID of *G. barbadense* was converted to that of *G. hirsutum* following gene homology.

### Phospho‐Proteome Analysis

The service for phospho‐proteome was provided by Novogene Co, Ltd. (Beijing, China) using an EASY‐nLCTM 1200 UHPLC system (Thermo Fisher, MA, USA) coupled with an Orbitrap Q Exactive HF‐X mass spectrometer (Thermo Fisher, MA, USA). The raw data of mass spectrometry was directly imported into the Proteome Discoverer 2.2 software (Thermo Fisher) for the identification and quantification of phosphorylated peptide and protein. For protein identification, protein with at least 1 unique peptide was identified at FDR less than 0.01 on peptide and protein level, respectively. Proteins containing similar peptides and could not be distinguished based on LC‐MS/MS analysis were grouped separately as protein groups. Precursor quantification based on intensity was used for Label‐free quantification. The protein quantitation results were used to screen the differentially expressed proteins by DEqMS.^[^ ^68^ ^]^

### Statistical Analysis

All experiments were performed with at least three independent biological replicates in this study. The pre‐processing of data and sample size (n) for each statistical analysis are listed in the figure legends. Error bars in the figures represent the standard deviation (SD) of the mean. Statistical analysis was performed using the Stst 1.0 software. The two‐tailed paired Student's *t* test was used to determine the significant difference between the two groups, and one‐way ANOVA test was used to determine the significant difference among more than two groups. Asterisks (∗) and letters (a/b/c) indicate statistical significance at *P* < 0.05, and double asterisks (∗∗) at *P* < 0.01.

## Conflict of Interest

The authors declare no conflict of interest.

## Author Contributions

X.M. and W.L. contributed equally to this work. W.G. conceived this project and designed all experiments. X.M. and W.L. performed the experiments. C.C., G.W., H.X., D.Z., and X.J. participated in the experiments. X.M. and W.L. wrote the manuscript. W.G. revised the manuscript. All authors discussed results and commented on the manuscript.

## Supporting information

Supporting Information

Supplemental Data 1

Supplemental Data 2

Supplemental Data 3

Supplemental Data 4

Supplemental Data 5

Supplemental Data 6

Supplemental Data 7

Supplemental Data 8

## Data Availability

The data that support the findings of this study are available in the supplementary material of this article.
