# Supplementary material for: GhMPK9‐GhRAF39_1‐GhWRKY40a Regulates the GhERF1b‐ and GhABF2‐Mediated Pathways to Increase Cotton Disease Resistance
Source: Adv Sci (Weinh). 2024 Jun 6;11(29):2404400. doi: 10.1002/advs.202404400 (PMC11304259; doi:10.1002/advs.202404400)
Supplement: Supplementary file 1 — Supporting Information [file ADVS-11-2404400-s003.pdf]

ADVANCED SCIENCE Open Access

Supporting Information

for *Adv. Sci.*, DOI 10.1002/advs.202404400

GhMPK9-GhRAF39_1-GhWRKY40a Regulates the GhERF1b- and GhABF2-Mediated Pathways to Increase Cotton Disease Resistance

*Xinyue Mi, Weixi Li, Chuan Chen, Huijuan Xu, Guilin Wang, Xuanxiang Jin, Dayong Zhang and Wangzhen Guo**

Supplemental information

GhMPK9-GhRAF39_1-GhWRKY40a Regulates the *GhERF1b*- and *GhABF2*-mediated Pathways to Increase Cotton Disease Resistance

Xinyue Mi#, Weixi Li#, Chuan Chen, Huijuan Xu, Guilin Wang, Xuanxiang Jin, Dayong Zhang, Wangzhen Guo*

Corresponding author:

Wangzhen Guo (moelab@njau.edu.cn);

This PDF file includes:

Supplemental Figures S1 to S8

Supplemental Tables S1 to S2

Supplemental Figures

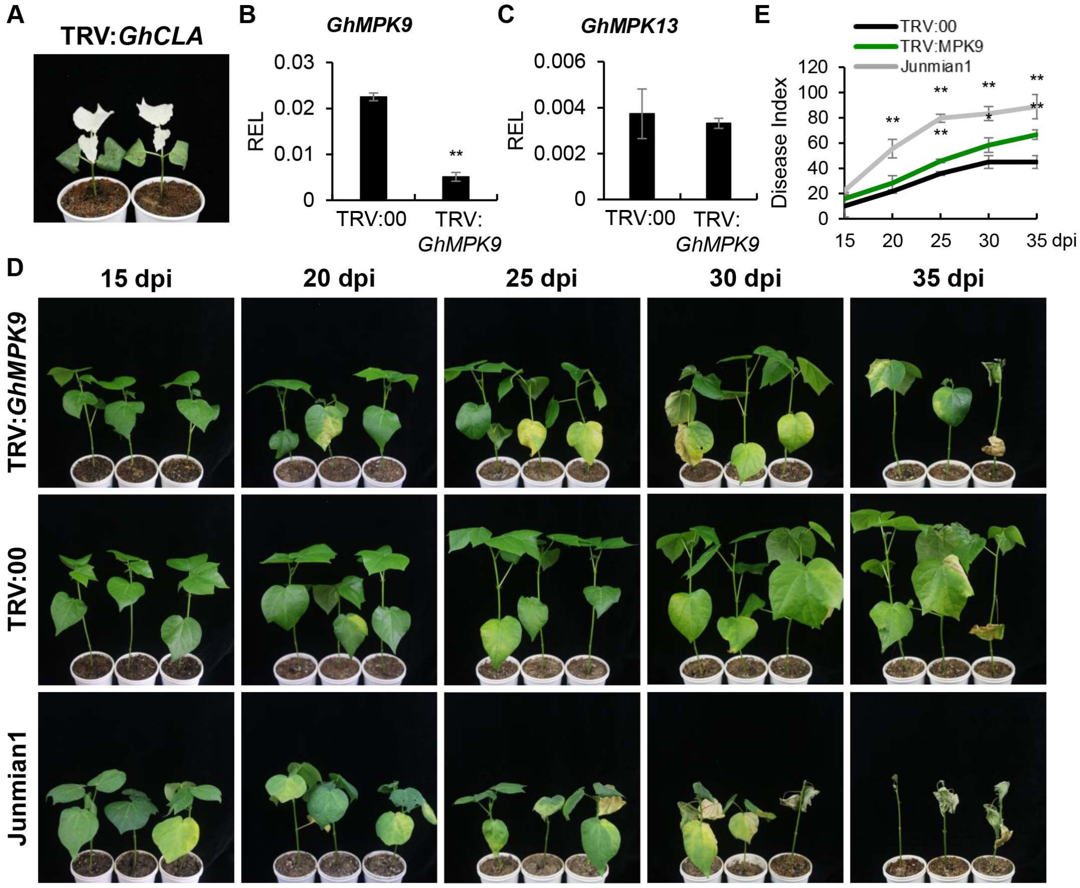

Figure S1. The roles of *GhMPK9* in the cotton defense response to Verticillium wilt.

(A) Phenotypes of TRV:*GhCLA* seedlings on 14th day post VIGS (dpv).

(B-C) The relative expression level (REL) of *GhMPK9* and *GhMPK13* in TRV:00 and TRV:*GhMPK9* seedlings on 14 dpv. The values are normalized to those of *Histone3*.

(D) Phenotypes of TRV:00, TRV:*GhMPK9* and Junmian1 (susceptible control) seedlings from 15th day post V991 inoculation (dpi) to 35 dpi.

(E) The disease index of TRV:00, TRV:*GhMPK9* and Junmian1 during 15 to 35 days after V991 inoculation.

Data are presented as means ± SD in (B, C and E), n = 9 (B and C), n = 30 (E), statistical analyses were performed using Student's *t* test: *, $P < 0.05$ and **, $P < 0.01$.

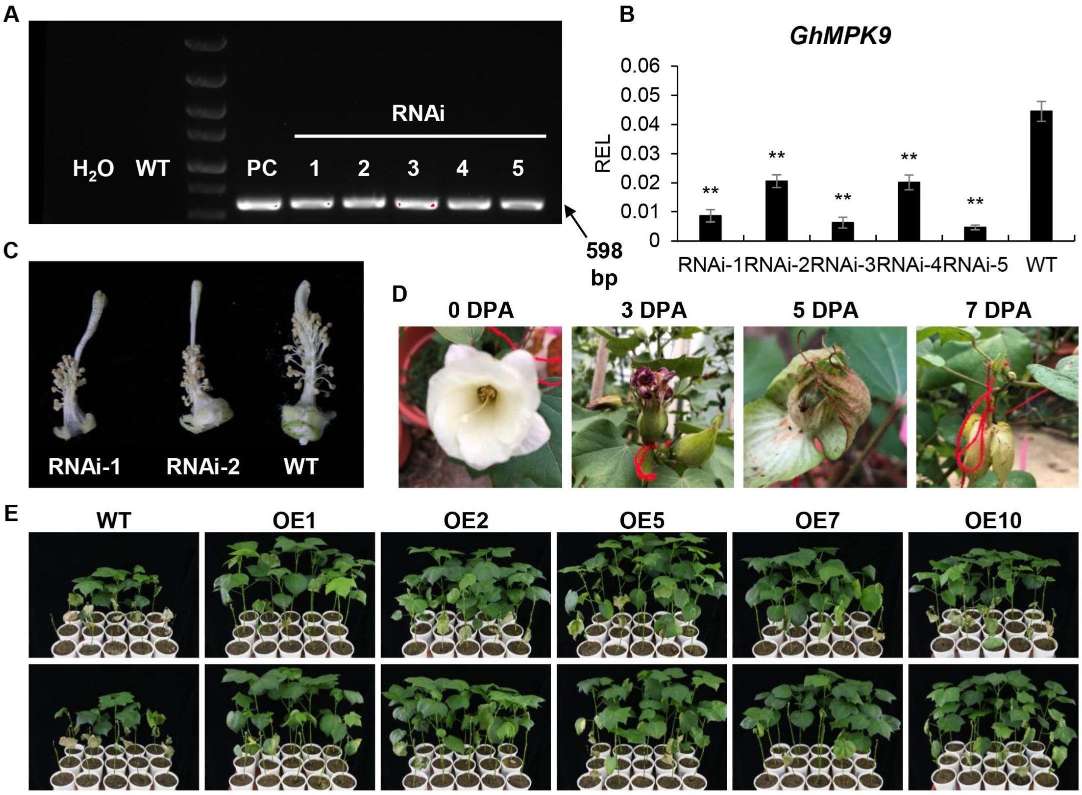

Figure S2. The phenotype observation of *GhMPK9*-RNAi and *GhMPK9*-OE transgenic plants.

(A) DNA detection of *GhMPK9*-RNAi lines by PCR analysis. PC: positive control; WT: wild-type; RNAi: *GhMPK9* suppression transgenic lines.

(B) The relative expression level (REL) of *GhMPK9* in wild-type and transgenic plants. Expression levels were normalized to those of *Histone3*.

(C) The phenotype of stigma, stamen and pistil in wild-type and transgenic cotton plants (RNAi-1 and RNAi-2).

(D) The phenotype of boll after pollination in *GhMPK9*-RNAi transgenic cotton. At 7-day post anthesis after pollination, the ovules fall off.

(E) The phenotype of leaf in *GhMPK9*-OE transgenic cotton at 30- day post V991 inoculation. OE: *GhMPK9* overexpression transgenic lines. Each OE line contains 30 plants.

Data are presented as means ± SD in (B), n = 9, statistical analyses were performed using Student's *t* test: **, $P < 0.01$.

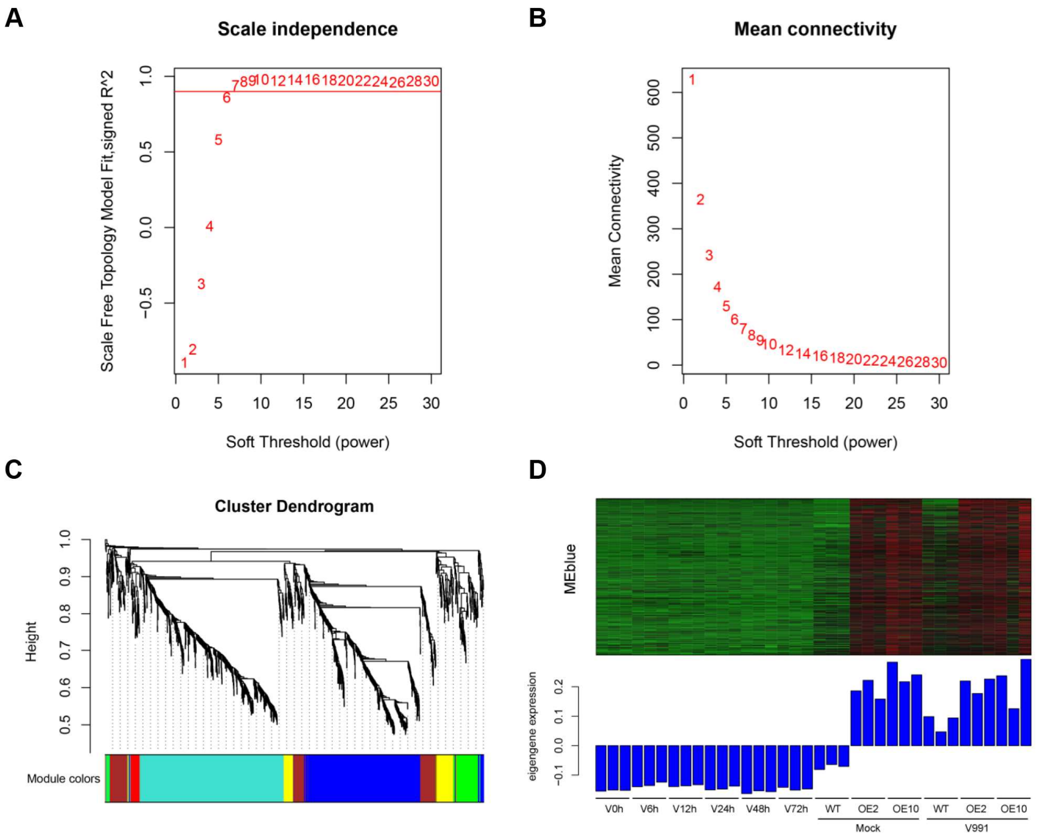

Figure S3. Weighted correlation network analysis (WGCNA) for the overlapped 1458 DEGs.

(A-B) The identification of soft threshold (power) in WGCNA. The horizontal axis represents weight parameters β. (A) The vertical axis on the left represents the square of the correlation coefficient between log(k) and log(p(k)) in the corresponding network. (B) The vertical axis on the right represents the mean of all gene adjacency functions in the corresponding gene module.

(C) Gene clustering dendrogram obtained by hierarchical clustering of adjacency-based dissimilarity. The color row below the dendrogram indicates module membership. Gray color denotes the genes unclassified into any module.

(D) The upper row shows the heatmap of the MEblue module genes across the transcriptome. The lower row shows the corresponding module eigengene (ME) expression values (y-axis) versus the same samples. Note that the ME takes on low values in samples where a lot of module genes are under-expressed (green color in the heatmap). Conversely, the ME takes on high values where module genes are over-expressed (red in the heatmap).

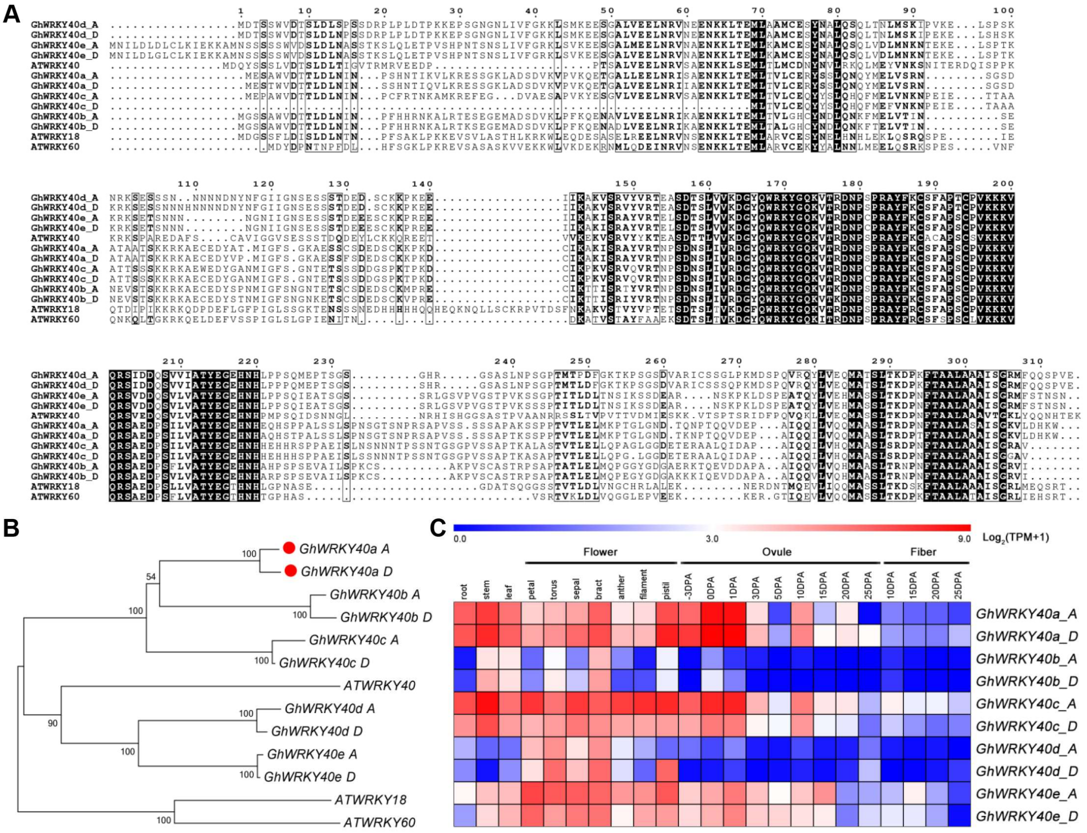

Figure S4. Structural and expression characteristics of *GhWRKY40s*.

(A) Alignments of protein sequences among AtWRKY40, AtWRKY18, AtWRKY60 and their homologous genes in cotton. There are two copies in tetraploid cotton genome for each pair, with each one in A and D subgenome, respectively.

(B) The phylogenetic tree of *AtWRKY40*, *AtWRKY18*, *AtWRKY60* and five pairs of homologs of *GhWRKY40* (a-e).

(C) The expression patterns in different tissues and organs for five pairs of homologs of *GhWRKY40* (a-e). Colored squares indicate expression levels of the selected genes from 0 (blue) to 9 (red) normalized by Log_2 (TPM+1).

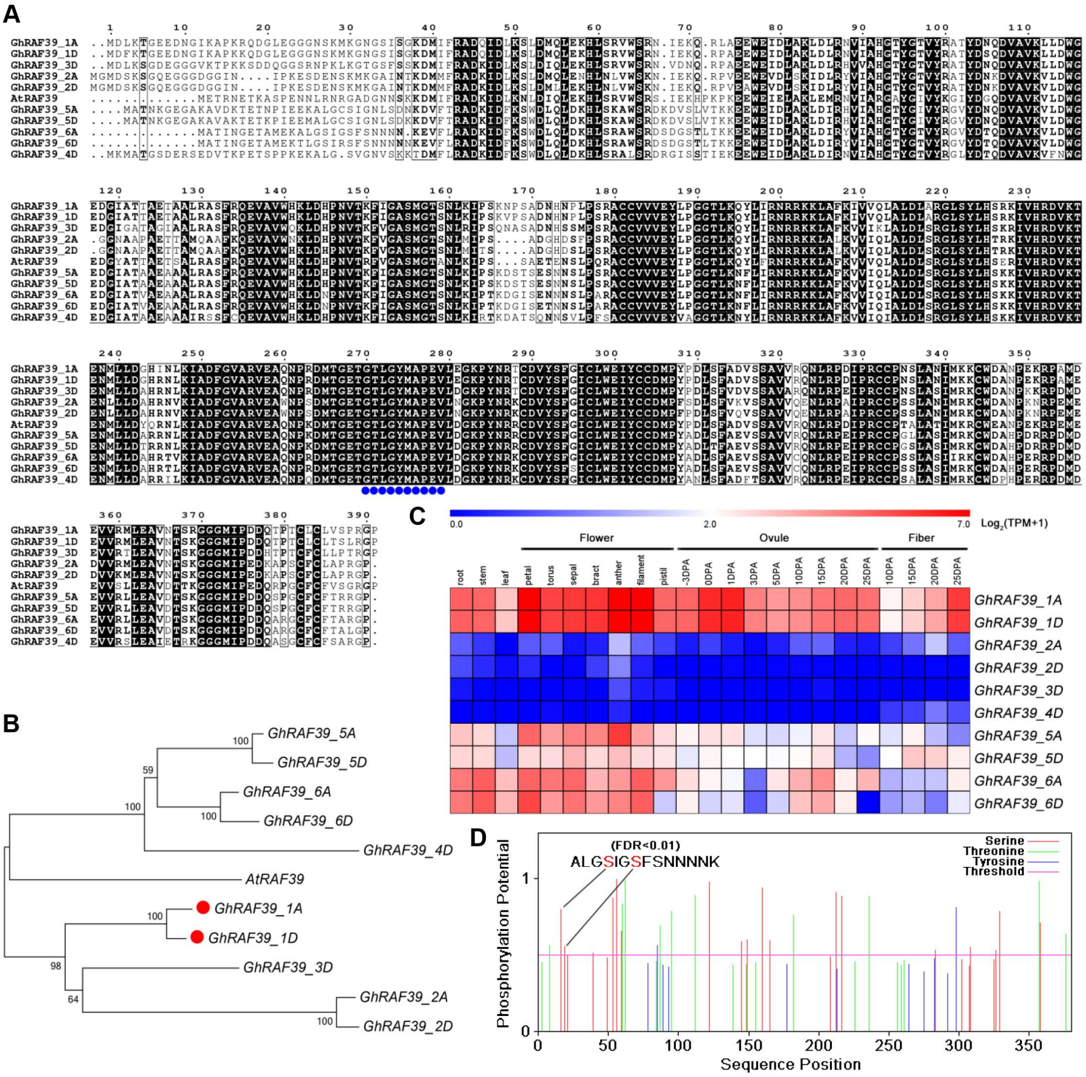

Figure S5. Structural and expression characteristics of *GhRAF39s*.

(A) Alignments of protein sequences among AtRAF39 and its homologous genes in cotton.

(B) The phylogenetic tree of *AtRAF39* and six pairs of homologs of *GhRAF39* (1-6). *GhRAF39-3* and *GhRAF39-4* have only one copy in D subgenome, others have two copies with each one in A and D subgenome, respectively.

(C) The expression patterns in different tissues and organs for six pairs of homologs of *GhRAF39* (1-6). Colored squares indicate expression levels of the selected genes from 0 (blue) to 7 (red) normalized by Log_2 (TPM+1).

(D) The prediction analysis of phosphorylation sites in GhRAF39 phospho-peptides.

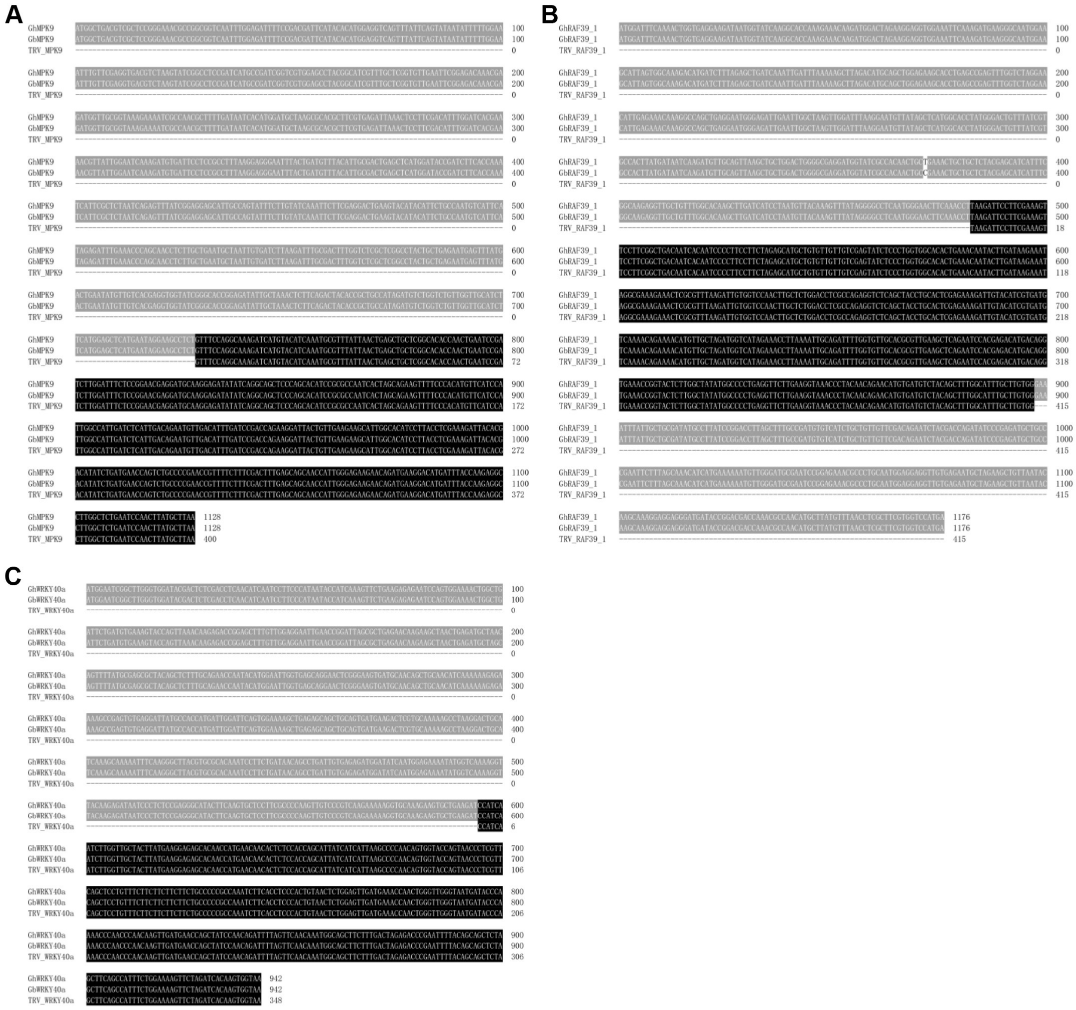

Figure S6. Sequence alignments of *MPK9* (A), *RAF39_1* (B) and *WRKY40a* (C) between *G. hirsutum* and *G. barbadense* with their corresponding silenced fragment in VIGS experiment. The identical sequences are shaded in black.

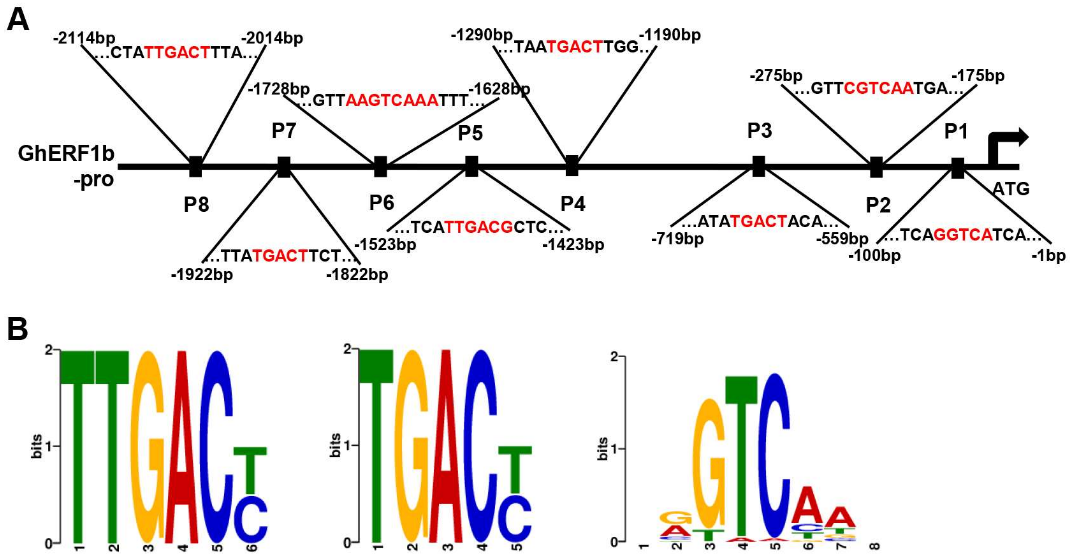

Figure S7. The predicted binding sites of GhWRKY40a in the promoter region of *GhERF1b*.

(A) Schematic diagram of W-box binding sites contained in *GhERF1b*-promoter region. The promoter was divided into eight segments (P1-P8). W-box motifs are labeled in red.

(B) The predicted W-box motifs in the *GhERF1b*-promoter.

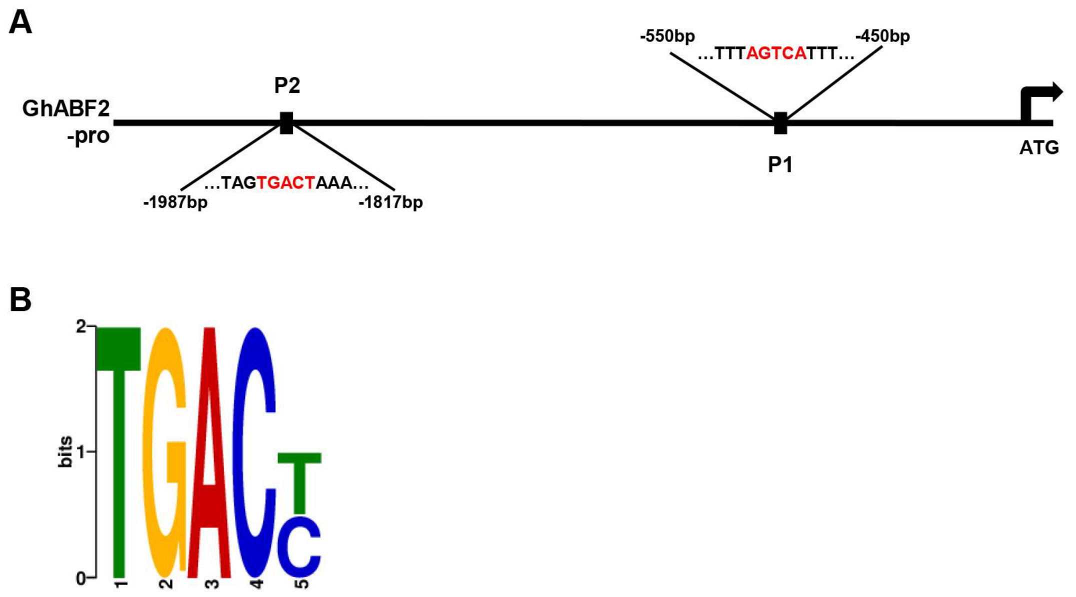

Figure S8. The predicted binding sites of GhWRKY40a in the promoter region of *GhABF2*.

(A) Schematic diagram of W-box binding sites in *GhABF2*-promoter region. The promoter was divided into two segments (P1, P2). W-box motifs are labeled in red.

(B) The predicted W-box motif in the *GhABF2*-promoter.

Supplemental Tables

Table S1. Field phenotype analysis of fiber quality and yield components in wild-type and *GhMPK9* overexpressing transgenic lines.

Fiber Traits	WT	OE2	OE10
Fiber length (mm)	28.3±1.61	28.93±0.68	28.26±0.48
Fiber strength ($cN\cdot tex^{-1}$)	30.02±2.41	32.6±0.34	30.97±1.81
Micronaire	4.62±0.278	4.23±0.17*	4.18±0.14*
Fiber uniformity (%)	82.67±1.96	83.67±0.15	82.53±1.4
Boll weight (g)	4.1±0.44	4.59±0.22	4.38±0.32
Lint percentage (%)	38.04±0.64	38.48±1.07	38.24±1.54
Lint index (g)	6.47±0.34	6.39±0.21	6.4±0.88
Seed index (g)	10.53±0.28	10.22±0.67	10.32±1.13

**In contrast to WT, Student's t test: $P < 0.05$.*

Table S2. The primers used in this study.

Gene name	Forward primer (5'-3')	Reverse primer (5'-3')
GhMPK9	TAAGAAGCGAAAACCCGAGT	TCTTTCAATCCGCAATCCAC
GhMPK9-qRT-PCR	TGGGAGAAGAACAGATGAAG G	TGTATGAGCCACCACAAGGTAT
GhMPK9-TRV2	GGTCTAGATCCGACGATTCATA CACATG	CCTCGAGTAGCATTCAGCAAG AGGTTG
GhMPK9-pART27-1	TTTGGAGAGGACACGCTCGAG GCCTCTGTTTCCAGGCAAAGA	ACCAATTGGGGTACCGAATTC AAAGAAAATGGTTCGGGGCA
GhMPK9-pART27-2	TCATTAAAGCAGGACTCTAGA GCCTCTGTTTCCAGGCAAAGA	GAAATCGATAAGCTTGGATCC AAAGAAAATGGTTCGGGGCA
GhMPK9-pCAMBIA 2301	GCTCTAGAATGGCTGACGTTG CTC	CGCCCGGGTTAATGATGATGAT GATGATGAGCATAAGTTGGATT TAGAGCC
GhMPK9-pGBKT7	ATGGCCATGGAGGCCGAATTC ATGGCTGACGTTGCTCCGG	CTAGTTATGCGGCCGCTGCAGT TAAGCATAAGTTGGATTTACAG CCA
GhMPK9-nYFP	GAGAACACGGGGGACTCTAGA ATGGCTGACGTCGCTCCG	GACAGTACTATCGATGGATCCA GCATAAGTTGGATTCAGAGCC A
GhMPK9-cYFP	ATTTACGAACGATAGTTAATTA AATGGCTGACGTTGCTCCGG	ACTGCCACCTCCTCCACTAGTA GCATAAGTTGGATTTACAGCCA A
cLUC-GhMPK9	TACGCGTCCCGGGGCGGTACC ATGGCTGACGTTGCTCCGG	TGTAGTCCATTTGTTGGATCCT TAAGCATAAGTTGGATTTACAG CCA
GhMPK9-pBINGFP4	TTACGAACGATAGCCGGTACC ATGGCTGACGTTGCTCCGG	GCCCTTGCTCACCATCCCGGG AGCATAAGTTGGATTTACAGCC AA
GST-GhMPK9	GATCTGGTTCCGCGTGGATCCA TGGCTGACGTTGCTCCGG	CTCGAGTCGACCCGGGAATTC TTAAGCATAAGTTGGATTTACA GCCA
His-GhMPK9	GCCATGGCTGATATCGGATCCA TGGCTGACGTTGCTCCGG	TTGTCGACGGAGCTCGAATTC TTAAGCATAAGTTGGATTTACA GCCA
MBP-GhMPK9	GAGGGAAGGATTTCAGAATTC ATGGCTGACGTTGCTCCGG	CAGGTCGACTCTAGAGGATCC TTAAGCATAAGTTGGATTTACA GCCA
GhMPK13-qRT-PCR	GGGAGAAGAGCAGATGAAGG	AGAAATGGCGAAAGCACAG
GhMPK3-qRT-PCR	TGCCATCTATTCGTCGATCG	GCCATTACAAACACCAACAG
GhMPK6-qRT-	AGAGGCTGAGTTGGAGTT	TAAAGGGTGGACATTTGGAAA

PCR		
GhRAF39_1-pGADT7	GCCATGGAGGCCAGTGAATTCATGGATTTCAAAACTGGTGAGGA	CAGCTCGAGCTCGATGGATCCTCATGGACCACGAAGCGAGG
GhRAF39_1-pGBKT7	AGGCCGAATTCCCGGGGATCCATGGATTTCAAAACTGGTGAGGA	CTAGTTATGCGGCCGCTGCAGTCATGGACCACGAAGCGAGG
GhRAF39_1-cYFP	GAGAACACGGGGGACTCTAGAATGGATTTCAAAACTGGTGAGGA	GACAGTACTATCGATGGATCCTGGACCACGAAGCGAGGTT
GhRAF39_1-nYFP	ATTTACGAACGATAGTTAATTAAATGGATTTCAAAACTGGTGAGGA	ACTGCCACCTCCTCCACTAGTTGGACCACGAAGCGAGGTT
GhRAF39_1-nLUC	GAGCTCGGTACCCGGGGATCCATGGATTTCAAAACTGGTGAGGA	CGCGTACGAGATCTGGTCGACTGGACCACGAAGCGAGGTT
cLUC-GhRAF39_1	TACGCGTCCCGGGGCGGTACCATGGATTTCAAAACTGGTGAGGA	TGTAGTCCATTTGTTGGATCCTCATGGACCACGAAGCGAGG
GhRAF39_1-pBINGFP4	TTACGAACGATAGCCGGTACCATGGATTTCAAAACTGGTGAGGA	GCCCTTGCTCACCATCCCGGGTGGACCACGAAGCGAGGTT
GhRAF39_1-TRV2	GTGAGTAAGGTTACCGAATTCTAAGATTCCTTCGAAAGTTCCTTCG	CGTGAGCTCGGTACCGGATCCCCACAAGCAAATGCCAAAGC
GhRAF39_1-qRT-PCR	TGCTGCGATATGCCTTAT	CTCACAACCTCCTCCATT
GhRAF39_1-GST	GATCTGGTTCCGCGTGGATCCATGGATTTCAAAACTGGTGAGGA	CTCGAGTCGACCCGGGAATTCTCATGGACCACGAAGCGAGG
GhRAF39_1-pBI121	GCAGGGGACTCTAGAGGATCCATGGATTTCAAAACTGGTGAGGA	ATCGGGGAAATTCGTGAGCTCTCACTTATCGTCGTCATCCTTGTAATCTGGACCATGGACCACGAAG
GhWRKY40a-qRT-PCR	AATCTTGGTTGCTACTTATGAA	GTTACAGTGGGAGGTGAA
GhWRKY40a-nLUC	GAGCTCGGTACCCGGGGATCCATGGAATCGGCTTGGGTGG	CGCGTACGAGATCTGGTCGACCCACTTGTGATCTAGAACTTTTCCAG
GhWRKY40a-pGADT7	GCCATGGAGGCCAGTGAATTCATGGAATCGGCTTGGGTGG	CAGCTCGAGCTCGATGGATCCTTACCACTTGTGATCTAGAACTTTTCC
GhWRKY40a-cYFP	ATTTACGAACGATAGTTAATTAAATGGAATCGGCTTGGGTGG	ACTGCCACCTCCTCCACTAGTCCACTTGTGATCTAGAACTTTTC

		CAG
GhWRKY40a-pBINGFP4	TTACGAACGATAGCCGGTACCATGGAATCGGCTTGGGTGG	GCCCTTGCTCACCATCCCGGGCCACTTGTGATCTAGAACTTTTCCAG
GhWRKY40a-TRV2	GTGAGTAAGGTTACCGAATTCCCATCAATCTTGGTTGCTACTTATG	CGTGAGCTCGGTACCGGATCCTTACCACTTGTGATCTAGAACTTTTCC
His-GhWRKY40a	GCCATGGCTGATATCGGATCCATGGAATCGGCTTGGGTGG	TTGTCGACGGAGCTCGAATTCTTACCACTTGTGATCTAGAACTTTTCC
GhWRKY40a-pBI121	ACGGGGGACTCTAGAGGATCCATGGAATCGGCTTGGGTGG	CGATCGGGGAAATTCGAGCTCTCACTTATCGTCGTCATCCTTGTAATCCCACTTGTGATCTAGAACTTTTCCAG
GhERF1b-qRT-PCR	TCCGATAACTCTAAGACGAAT	GCCTAACGCCTCTGTAAT
GhERF1b-TRV2	GTGAGTAAGGTTACCGAATTCATGGAGATGTATTCAAGTGCAAACG	TCCCCATGGAGGCCTTCTAGAGGCACCACCGCAGGAACC
GhERF1b-LUC	TCGACGGTATCGATAAGCTTGTCAGGTCATCACGACCGTACATCATTGTATCAA	GCTCTAGAACTAGTGGATCCATCGCTGAGTAAGTATTGCCGAATTGAGTCCAG
GhERF1b-1-pAbAi	AAATGATGAATTGAAAAGCTTGTCAGGTCATCACGACCGTACA	GTCGACAGATCCCCGGGTACCAAATCGATGATGAGGACCAAGC
GhERF1b-2-pAbAi	AAATGATGAATTGAAAAGCTTCCATCTTCAGGGCATAGAGTCTTT	GTCGACAGATCCCCGGGTACCACCGCGTCCGGTTTGTGG
GhERF1b-3-pAbAi	AAATGATGAATTGAAAAGCTTTCCTGATGTGAAAGATCAGACAAAG	GTCGACAGATCCCCGGGTACCTCACTTTATTTTTCGTTCCGGTC
GhERF1b-4-pAbAi	AAATGATGAATTGAAAAGCTTTAAATTATATGGTGTAGCTAGAGCAGTAATG	GTCGACAGATCCCCGGGTACCAAAGTATTAAAGAGGTTAAATTGAAATGTTAA
GhERF1b-5-pAbAi	AAATGATGAATTGAAAAGCTTAAAAGTTTTTTTTCAATATTTTTTCGC	GTCGACAGATCCCCGGGTACCCAATGAAGAAAAAGTGAAAAATATTTAGAA
GhERF1b-6-pAbAi	AAATGATGAATTGAAAAGCTTTTTATATATTTATAGTTTTTTAAAGAGTTAAGTCAAA	GTCGACAGATCCCCGGGTACCATTTATAAAATTTTAAATTAGTAATAATAAAATTATATTTT
GhERF1b-7-pAbAi	AAATGATGAATTGAAAAGCTTAAACATTTAATTTTATGTTTGCTCCC	GTCGACAGATCCCCGGGTACCTTAATTTAATTAATCTTGTGGCTTAAACA

GhERF1b-8-pAbAi	AAATGATGAATTGAAAAGCTTTTCCGTGCCATAATATTCTTTAATG	GTCGACAGATCCCCGGGTACCTGGCCCTAGACGTATTATATAAAAGTTAA
ProGhERF1b-EMSA	AATATTCTTTAATGGACTATTGACTTTATTCCGAACTTATCCAAAAGGTT	AACCTTTTGGATAAGTTCGGAATAAAGTCAATAGTCCATTAAAGAATATT
GhABF2-qRT-PCR	TTCAGGGTGGAAAGATGG	GCGAGACAGAGGAAGTAT
GhABF2-TRV2	GTGAGTAAGGTTACCGAATTCAAGATATTTCAAAGGAGTATTCAATAGGG	TCCCCATGGAGGCCTTCTAGAGGTGGTCCCTGATCCCCC
GhABF2-LUC	GTCGACGGTATCGATAAGCTTAAAAACGTATAAATGTGTAACAAAGATTATAA	CGCTCTAGAACTAGTGGATCCAAGTATGGGCAAGAACTAGACAAAAC
GhABF2-1-pAbAi	AAATGATGAATTGAAAAGCTTATTAAAATAGTTATTTTTGTTTATCTTAAATTACAT	GTCGACAGATCCCCGGGTACCCTTACCATTATACTGTTAACGGCAATT
GhABF2-2-pAbAi	AAATGATGAATTGAAAAGCTTAGCTTATTGTTACACTGTTAACGGAAA	GTCGACAGATCCCCGGGTACCGAACTAGACAAAACTTTTTAGGTAAACTATCA
ProGhABF2-EMSA	GGCTTAGTGACTAAAATGTTACAACACGATAACGTAAGTGACTAAAACGT	ACGTTTTAGTCACTTACGTTATCGTGTTGTAACATTTTAGTCACTAAGCC
GhPR1-qRT-PCR	AAGAATGTGGGTTAGTGAGAGGGT	ACCACTTGAGTATAATGCCCGC
GhPR2-qRT-PCR	CCACCAGCAGCAGAAGTTATCG	TTCAAGGTTTGCACTCGGAAGA
GhPR3-qRT-PCR	ACTCCACAATCACCGAAGCCAT	GCATTCCAACCCTTACCACATTC
GhPR4-qRT-PCR	TTGCGGCAATGGCTTCAATC	TGCTCTCACATTATTCGGCA
GhPR5-qRT-PCR	GCCGTGATTCATACAGTTATCCTCA	TTGGCTCTTACTTCCGACCATCT
GhPR6-qRT-PCR	CTGGGTGTCCTGGGAAGAAC	TTGTAGGGGGACGAACAACG
GhEF-1α	AGACCACCAAGTACTACTGCAC	CCACCAATCTTGTACACATCC
Histone3	GAAGCCTCATCGATACCGTC	CTACCACTACCATCATGG
